# Behaviour change techniques used in interventions targeting dementia risk factors amongst older adults in rural and remote areas: A systematic review and meta-analysis

**DOI:** 10.1016/j.tjpad.2025.100093

**Published:** 2025-02-22

**Authors:** Laura Dodds, Kay Deckers, Celia B. Harris, Joyce Siette

**Affiliations:** a The MARCS Institute for Brain, Behaviour and Development, Western Sydney University, Westmead, NSW 2145, Australia; b Alzheimer Center Limburg, Mental Health and Neuroscience (MHeNs) Research Institute, Department of Psychiatry and Neuropsychology, Maastricht University, the Netherlands

**Keywords:** Dementia, Risk reduction, Rural, Remote, Intervention

## Abstract

Behavioural interventions targeting health risk factors within rural areas are often not tailored to effectively address the needs and socio-environmental barriers to access and behaviour change faced by these communities. Little is known about the underlying behaviour change mechanisms that contribute to reducing dementia risk for communities living in regional and rural areas. This systematic review and meta-analysis aimed to summarise the effectiveness of behavioural interventions targeting late-life single modifiable dementia risk factors (physical inactivity, poor diet, social isolation and depression) and the mechanisms used to contribute to behaviour change. Six databases were searched to identify regional and rural behavioural interventions targeting modification of late-life dementia risk behaviours between 2000 and 2024. Behaviour change techniques (BCTs) and outcomes for each intervention were extracted. Where possible, meta-analyses were performed to assess the effectiveness of the behavioural intervention on outcomes related to dementia risk. Out of 42,529 articles, 49 studies were included: 22 on physical inactivity, 6 on poor diet, 9 on social isolation, and 12 on depression. Many BCT categories were applied (M = 14.8, SD = 10), with high use of goals and planning (49/49 interventions; 100 %), shaping knowledge (47/49 interventions; 95.9 %), social support (43/49 interventions; 87.8 %) and comparison of outcomes (38/49 interventions; 77.6 %). Social isolation interventions used the most BCTs (M = 18.3; SD = 8.5), followed by depression (M = 17.6; SD = 10.7), physical inactivity (M = 16.0; SD = 11.5), and poor diet (M = 5.2; SD = 3.1). Although effectiveness was limited across interventions, apart from cognitive behavioural therapy for depression (SMD −0.39, 95 % CI −0.55 to −0.24), future programs targeting dementia risk factors would benefit from incorporation of BCTs. Simultaneously, consideration of the socio-environmental context, accessibility, and community involvement in rural and regional areas may improve the sustainability of interventions.

## 1 Introduction

The global population is ageing rapidly, and in many countries dementia prevalence is a persistent concern [1]. Consequently, prevention efforts are a priority for many national and international policy agendas. The World Health Organization (WHO) key action plan [2] and the Australian National Dementia Data Improvement Plan 2023–2033 [3] aim to aid governments in providing resources and guidelines to educate communities on ways to minimise their risk of dementia [2,4,5]. A key tenet of these plans is to promote evidence-based dementia research to develop cost-effective and sustainable interventions that target key modifiable dementia risk factors [6,7].

### 1.1 Current evidence for multidomain interventions and dementia risk factors

Modifiable risk and protective factors for dementia have been identified [8,9] as well as their relative contribution to lifetime dementia risk according to each life stage. Physical inactivity, depression and obesity, and high alcohol intake have important impacts in midlife whilst social isolation, air pollution and visual loss have impacts on dementia risk in later life [9]. There have been concerted efforts worldwide to investigate multidomain lifestyle interventions that address these dementia risk factors simultaneously (e.g., FINGER trial [10], Body Brain Life [11]). Many trials have demonstrated positive effects in modifying dementia risk [[12], [13], [14], [15]] with a meta-analyses indicating high certainty evidence that such interventions can reduce dementia risk and moderate evidence for improvements in cognition [16]. Despite their contributions to risk reduction, the extent to which multidomain interventions can impact cognitive outcomes remains inconclusive, with various trials showing no improvements in global and domain-specific cognition or incident dementia [16,17] (e.g., PreDIVA [18], MAPT [19]). Research has been heavily focused on multidomain interventions and their impact on cognition [16,17,20], which makes it challenging to draw conclusions about which multidomain program components are associated with any benefit. We therefore aimed to review the extent of single domain interventions and their impacts on behaviour change related to specific risk factors, particularly among individuals with multimorbidity [17,21].

### 1.2 Current evidence for multidomain interventions amongst at-risk communities

Variations in multidomain program findings might stem from intervention factors, as well as individual, environmental, and societal influences. Considering the nascent stage of this field, multidomain interventions for dementia have only emerged in the last decade [20], highlighting the need for additional exploration. There have been very few empirical multidomain dementia prevention efforts in populations that are at increased risk of dementia, such as in low- and middle-income countries (LMICs) [22], underserved communities [[23], [24], [25], [26]], and areas outside major cities [[27], [28], [29], [30], [31]]. Of the limited research available, a successful Balinese brain health education program adapted from a Japanese-style healthcare program has been implemented and found to improve physical fitness and brain function [32] and a coach-supported mHealth intervention (PRODEMOS) was moderately effective at reducing presence of risk factors in adults living in lower socioeconomic areas in the UK [12]. Many other preventive efforts for dementia and cognition are still in the developmental phase [24,30,33].

### 1.3 Socio-environmental context and tailoring risk reduction interventions for rural communities

At-risk populations often have a high prevalence of certain chronic conditions and dementia-related health risk factors such as physical inactivity [34,35], poor nutrition [36,37], obesity [38], smoking [39] and hypertension [38]. Currently, two-thirds of global dementia cases are within LMICs [40] and depending on the country, rural areas have a higher prevalence of dementia or are significantly disadvantaged by the cumulative effects of dementia and cognitive impairment [[41], [42], [43], [44]]. The design and implementation of public health interventions are predominantly based on the needs and socio-environmental context of metropolitan populations [28]. Whilst piloting interventions in these populations may produce favourable outcomes, there is a lack of consideration of the geographical isolation, limited transportation, reduced access to health services, health-care capacity issues, and community values and support that influence intervention outcomes to different extents depending on the rurality of specific contexts [39,45,46]. These challenges can often create resistance and hesitation towards interventions encouraging behaviour change and ultimately higher rates of morbidity, mortality and acute health care crises remain. Prioritising preventive interventions tailored to the needs of rural populations and extrapolating the mechanisms used within these interventions that lead to favourable outcomes is important for addressing these disparities and improving overall health outcomes, including reducing the risk of dementia.

### 1.4 Incorporating behaviour change theory for single domain risk reduction

**Fig. 1:**
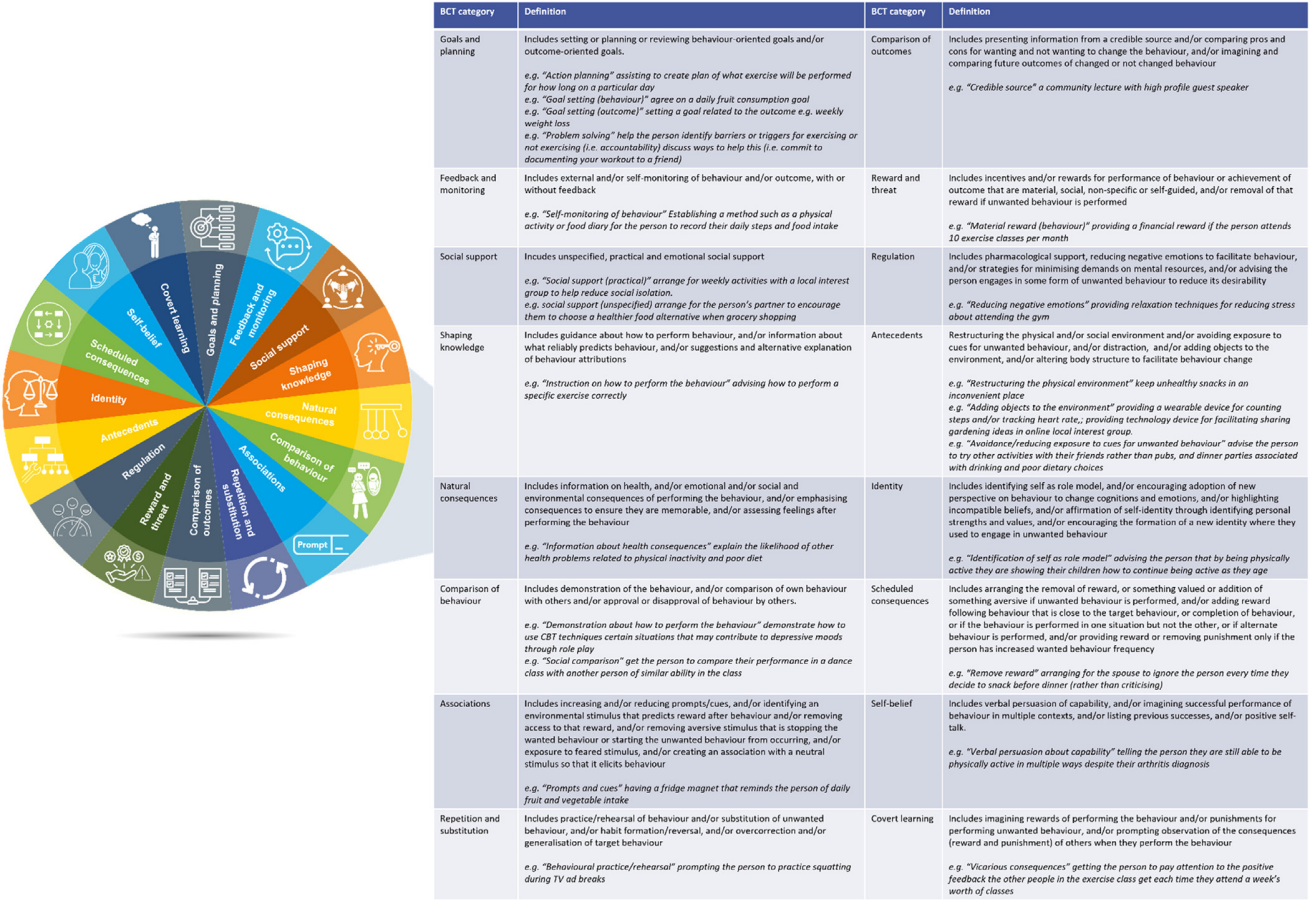
Behaviour change technique taxonomy (BCTTv1) categories adapted from Michie et al., The behaviour change technique taxonomy (v1) of 93 hierarchically clustered techniques: building an international consensus for the reporting of behaviour change interventions (2013) [49].

Behaviour change theory and implementation science are essential for understanding the mechanisms of any benefits associated with multidomain interventions [47]. Michie and colleagues [48] developed a taxonomy of 93 Behaviour Change Techniques (BCTs) organised into 16 categories as an ‘intervention design tool’ to help identify what intervention functions are likely to be effective in changing behaviour [49] (Fig. 1). Appendix I provides a link to Michie and colleagues full list of BCTs, BCT definitions and examples [49]. For example, action planning is a BCT found in the goals and planning category. Practical application of this BCT may be planning to attend an exercise class, at a specific time and an agreed number of days per week. Further, credible source is a BCT within the comparison of outcomes category and could include a presentation of information given by a well-known professional on depression and how to adopt CBT techniques to emphasise their effectiveness and importance in recovery. Mechanisms for behaviour change have been tested for several outcomes related to modifiable risk factors for dementia including physical inactivity [50,51], weight management [51,52], dietary intake [51,53], and depression [54,55]. Recent reviews suggest that isolated BCTs and certain combinations of BCTs can increase the efficacy of interventions, although findings remain mixed for different health conditions. For example, “goal setting” and “feedback on behaviour” were most effective for promoting physical activity amongst overweight and obese adults in the long-term [51]; “habit formation”, “salience of consequences” (i.e., making individuals pay more attention to information about consequences of behaviour) and “adding objects to the environment” (i.e., providing items that will help facilitate behaviour) were effective in improving dietary intake [53] and weight management [52]; providing education about health benefits of behaviour, instruction and demonstration, “action planning” or intention formation (i.e., encouraging people to decide to change or set a goal), and “social comparison” (i.e., sharing information about another person's performance and comparing it with their own experience) were effective in improving depression outcomes [54,55].

Despite the opportunity to improve intervention effectiveness, BCTs adopted within multidomain lifestyle intervention studies for dementia prevention cannot necessarily be generalised to different sociodemographic groups or geographical contexts with disparate access to services and resources. To date, BCTs have not been systematically identified from interventions aimed at modifying dementia risk factors in older adults residing in rural and remote areas.

This systematic review and meta-analysis addressed this gap by examining interventions implemented in geographically isolated communities to target four modifiable dementia risk factors: physical inactivity, poor diet, social isolation and depression [9,56]. This review aims to i) identify effective behavioural interventions that target these four single modifiable health risk factors for dementia independently of each other; ii) classify the applied behaviour change techniques (BCTs) for each risk factor and iii) describe BCTs used in effective behavioural interventions in improving outcomes (reducing risk) in regional and rural communities.

## 2 Method

The planning and reporting for this review followed the Preferred Reporting Items for Systematic reviews and Meta-Analyses (PRISMA) guidelines [57] and was pre-registered on PROSPERO (CRD42021278021). A PRISMA checklist can be found in Appendix II.

### 2.1 Data sources and search strategy

The search strategy aimed to identify local, national and international behaviour change interventions or programs aimed at modifying late-life risk behaviours for dementia (i.e., physical inactivity, poor diet, social isolation and depression). It was developed by the research team in consultation with a clinical librarian and was undertaken in November 2021, with the search updated in November 2023. The six electronic databases included were MEDLINE, Embase, PsycINFO, Web of Science, Cumulated Index to Nursing and Allied Health Literature (CINAHL) and the Cochrane Library. To maintain consistency in the quality and relevance of included studies to current practices and technologies, we also limited the date range of our articles to 2000 onwards.

Search terms for each risk factor were combined with descriptors of middle aged and older adults (e.g., *aged* OR *elderly* OR older adult* OR *geriatric* OR *aged 80 and over* OR older senior* OR middle-age*), rural or underserved populations (e.g., *rural* OR *remote* OR *nonmetropolitan OR* underserved OR *region* OR province) and intervention-based search terms (e.g., program* OR intervention* OR behave* intervention OR health promotion*). An example of risk factor search terms for physical inactivity are *Exercise/* OR *Exercise movement techniques/* OR *Exercise therapy/* OR *Sedentary behaviour/* OR *physical activity* OR exercise* OR physical inactivity or sedentary behave* OR sport* or athletic participation OR exercise behave* OR walk* OR *fitness*. A complete outline of search terms including database specific MeSH terms and keywords/specifiers can be accessed in Appendix III.

### 2.2 Inclusion and exclusion criteria

A predefined eligibility criteria was generated to determine the inclusion of articles. Studies were included if they were (i) peer-reviewed articles, (ii) available in English language, (iii) used behaviour change techniques to target adults aged 55 years and over in a rural or remote setting, and (iv) evaluated the effectiveness of a program in improving a dementia risk factor of physical inactivity, poor diet, depression or social isolation. Categorisation of remoteness was defined based on country-specific population density. Age range was set at 55 years or older due to the lower life expectancy for older adults living outside of major cities [58].

We limited studies to interventions including RCTs, quasi-experimental, and mixed method studies that were targeting behavioural modification in relation to each risk factor, individually (e.g., studies with a main behaviour change component targeting physical inactivity, as well as a primary outcome measure of physical activity e.g. steps/day, physical function were included in studies targeting physical inactivity). Studies were still included if they measured secondary outcomes related to other risk factors like depression or social isolation. Interventions that used standardised assessments to measure risk factors or indicators of these risk factors were included (e.g., cardiovascular fitness as an indicator of physical activity), whereas qualitative study designs without objective outcome measures were excluded. Articles were also excluded if the intervention was pharmacological or delivered in a clinical or healthcare setting (e.g., hospital or primary care practice).

### 2.3 Study selection

All potential studies were exported into a reference manager (EndNote version 20) and duplicates were removed. A mobile and web-based application for systematic reviews (Rayyan) was used to screen titles and abstracts and identify studies that satisfied the eligibility criteria. The primary author (LD) removed duplicates and conducted initial screening of titles and abstracts for inclusion. At least 2 reviewers were assigned to each risk factor and 5 % blinded double-coding was conducted between each of the two reviewers assigned to that risk factor (LD, JS, VC, AM, MC, SMS, JWK, DP, EW, SW, TW) to ensure inter-rater reliability. The results were in high agreement ranging from 95 to 98 % for each set of articles included for each risk factor. Manual searching of reference lists for included articles was conducted. Selected full-text articles were then obtained for final screening completed by the primary author (LD) and independently assessed against the inclusion criteria by one reviewer (JS). In case of discrepancy between LD and JS, then discussion was conducted with CH.

### 2.4 Data extraction

Data extraction was completed by the primary author (LD) according to pre-defined data extraction fields and verified by an additional reviewer (JS). Study characteristics (e.g., author, year of publication, country), methodology (study design, intervention details, assessment tools used), recruitment and attrition rates, sample characteristics (e.g., sample size, age, gender), primary and/or secondary study outcomes (mean or mean differences before intervention and at first follow-up timepoint) and behaviour change techniques were extracted.

### 2.5 Coding behaviour change techniques

The primary interest of this review was the use of behaviour change techniques within interventions targeting dementia risk factors of physical inactivity, poor diet, social isolation and depression. Behaviour change techniques within each intervention were identified and categorised according to Michie et al.’s BCT taxonomy (v1) [49].

At least two coders independently coded all interventions using BCTv1 definitions and examples. Adopting the approach from Presseau [59] and Samdal [51] and colleagues, BCTs were coded if there was clear evidence that it had been used to target either physical inactivity, improvements in diet or food consumption, social isolation or depression depending on the risk factor the paper had been assigned to. Coders were experienced in applying BCT taxonomy and designing interventions within health services (KD), aged care (CH), gerontology (KD, JS) and public health (JS, LD). Throughout the initial coding process, coders updated the coding manual if further assumptions were made to provide clarity when discussing with the research team. After initial coding, a summary report was created using Excel to present BCTs by study and identify discrepancies to be discussed with the research team. Coding inconsistencies were reviewed and deliberated upon in discrepancy-resolution meetings to ensure agreement was reached and the coding manual updated.

### 2.6 Coding assumptions

BCTs were coded if they were used to alter the behaviour of participants in the intervention group. Two primary assumptions were made. Firstly, if specific behaviours were not clear, BCTs were coded at a general behavioural level and if certain elements within intervention provision that were mentioned without further details provided, we assumed the following BCT codes as a minimum for the specific intervention components. For example, education-focused interventions included *instruction on how to perform the behaviour;* training-targeted programs included *instruction on how to perform the behaviour* and Cognitive Behavioural Therapy included *action planning, instruction on how to perform the behaviour,* and *social support (emotional)*.

### 2.7 Analysis

Narrative and descriptive synthesis were conducted, where possible, to quantify the proportion of studies within each risk factor with BCTs coded in a specific category, the average number of BCTs coded across studies for each risk factor, as well as the most frequently used BCTs in interventions for each risk factor.

### 2.8 Risk of bias

The Cochrane Risk of Bias tool version 2 (RoB2) [60] was used to assess overall risk of bias for each study which used a randomised controlled trial design. The tool was independently completed by two researchers (VC and LD). Inter-rater reliability was moderately high with 76.5 % agreement between researcher decisions. Discrepancies were resolved through discussion and checked by an additional researcher (JS). For each of the 5 domains of bias, a quality rating of unclear, low, or high risk was determined using signalling questions. Research team provided comments for each domain as support for decisions made.

### 2.9 Certainty of the evidence

We used the Grading of Recommendation, Assessment, Development and Evaluations (GRADE) approach to rate the certainty of evidence in 5 areas. Apart from Risk of Bias, inconsistency, indirectness, imprecision and publication bias ratings were assessed based on the GRADE series guidelines [[61], [62], [63], [64], [65], [66]]. Reasons for each decision to downgrade are provided in Appendix IV.

### 2.10 Meta-analysis

Randomised controlled trials with similar interventions (behavioural interventions targeting rural, regional or remote adults over 55 years of age) and primary and/or secondary outcome data were combined using a random-effects model to pool effect sizes along with 95 % CIs using the RevMan Web 2020 computer program [67]. This model accounts for variations within study populations, methods and interventions implemented. Heterogeneity across studies was determined using the *I*^2^ statistic in which values above 50 % indicated substantial heterogeneity. Risk of small-study bias was assessed visually using Funnel plots (Appendix V). Outcomes for each factor are listed as: physical inactivity trials (physical function), social isolation (loneliness), depression programs (depression). Outcomes across RCTs within poor diet interventions were not homogenous and a meta-analysis could not be conducted. Standardised mean differences were selected as an alternative to simple mean differences, in light of the diversity in measures adopted in each study. These were used to calculate effect sizes (Cohen's d) for continuous outcomes using available means, standard deviations and sample sizes. Where data was not available authors were contacted, however if no response was provided within 1 month after 1 reminder email, this study was excluded from the meta-analysis.

## 3 Results

**Fig. 2:**
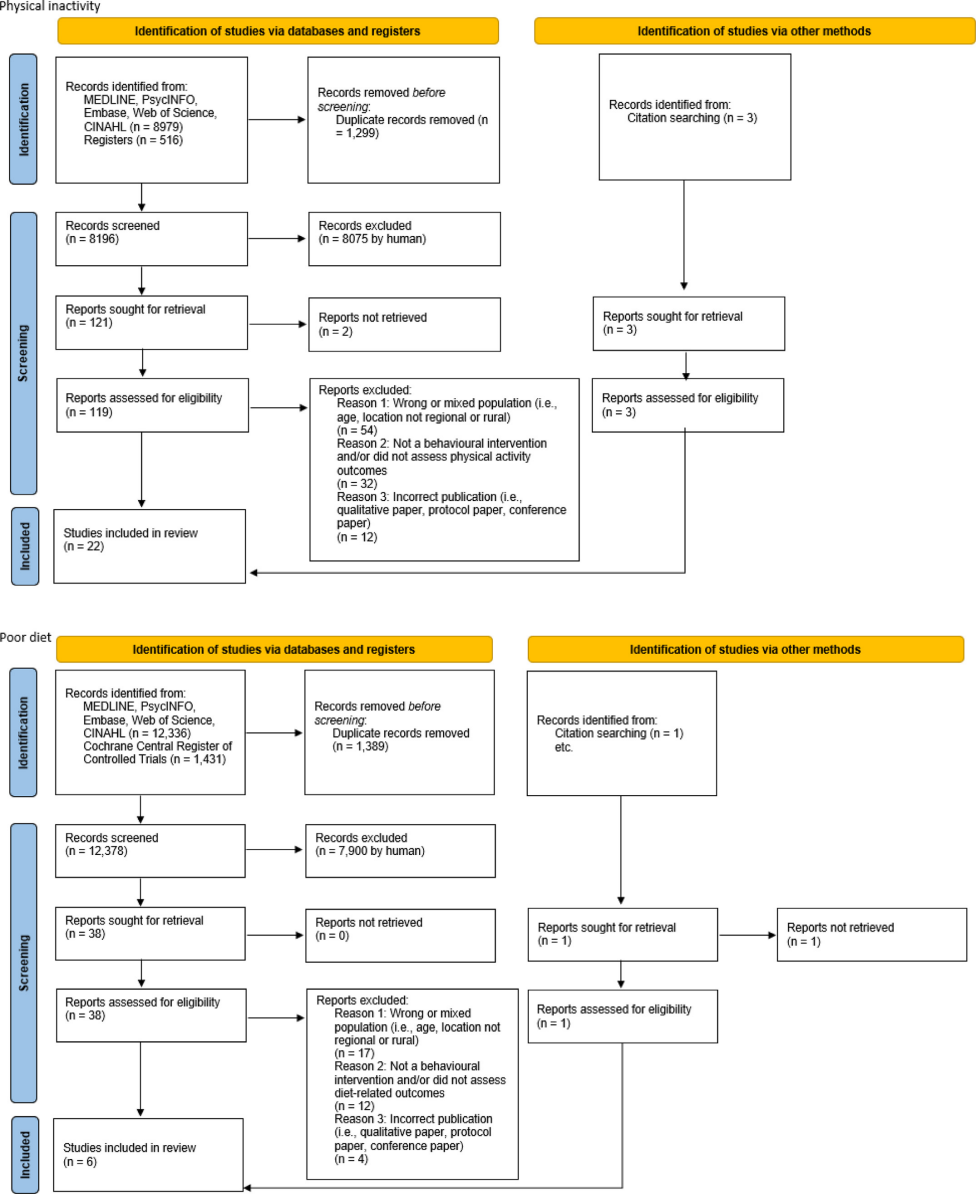

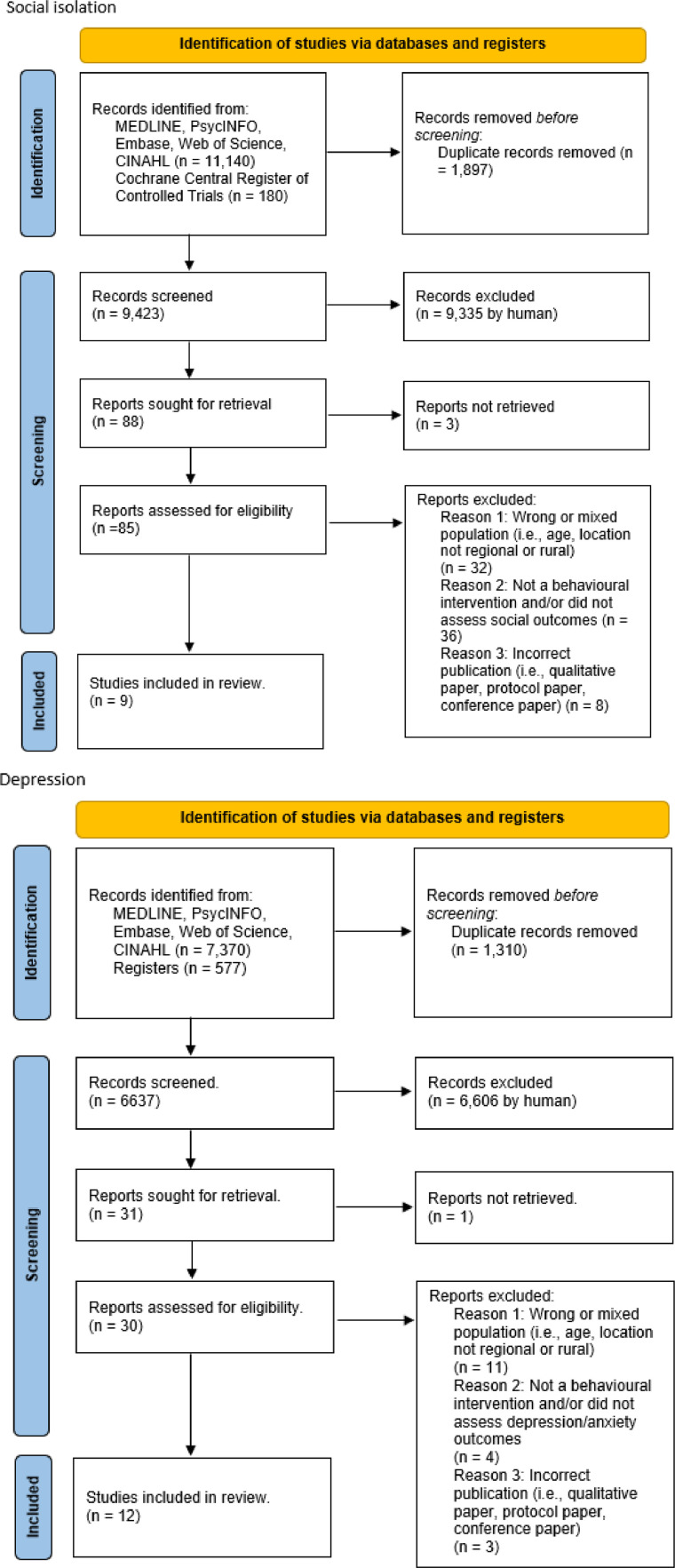
PRISMA flow diagrams for risk factors physical inactivity, poor diet, social isolation and depression.

From 42,529 records, 166 full-text articles were assessed for eligibility and 49 unique studies were identified (physical inactivity = 22, poor diet = 6, social isolation = 9, depression = 12). The PRISMA flow diagrams for each risk factor are provided in Fig. 2. Of the 49 studies, physical inactivity had 10 RCTs and depression had 8 RCTs, followed by social isolation and poor diet with 2 RCTs each. However, some RCT studies did not have comparable outcomes and thus did not proceed with a meta-analysis (e.g., physical inactivity [[68], [69], [70], [71]], poor diet [[72], [73], [74]], and depression [75]).

### 3.1 Study and intervention characteristics

Table 1 summarises the characteristics of each study (i.e., age, gender, sample size, attrition rate, data collection time points, and outcomes). Table 2 summarises intervention characteristics (i.e., intervention components, duration, format, and setting). A detailed summary of all outcomes for each intervention is available in Appendix VI.

**Table 1:** Summary of study characteristics.

	Author (year)	Country	Study design	Sample Size	Mean age (SD)	% Female	Attrition rate	Data collection points	Evidence of benefit
Physical inactivity (*N* = 22)	Akihiro et al. [76]	Japan	RCT	I: 23 C: 34	I: 73.6 (4.9) C: 74.2 (5.1)	I: 52.3 % C: 50.0 %	0 %	2	Y^short^
	Cai et al. [77]	China	RCT	I: 34 C: 30	66.9 (4.2) I: 65.8 (4.2) C: 68.0 (4.1)	63.9 % I: 55.6 % C: 72.2 %	I: 5.6 % C: 16.7 %	2	Y^short^
	Cruz-Ferreira et al. [78]	Portugal	RCT	I: 32 C: 25	I: 71.1 (3.9) C: 72.8 (4.5)	100 %	0 %	3	Y^short^
	Harris et al. [71]	UK	Mixed Methods; RCT	I: 150 C: 148	NA	I: 54 % C:53 %	6.0 %	3	Y^short/long^
	Jeon et al. [79]	Republic of Korea	RCT	I: 31 C: 31	I: 69.32 (4.46) C: 69.16 (4.05)	100 %	I: 11.4 % C: 11.4 %	2	Y^short^
	Johnson et al. [80]	Canada	RCT	I: 40 C: 29	81 (8.0) I: 80.7 (7.9) C: 79.7 (9.4)	83 % I: 85 % C: 79.3 %	24 % I: 27.5 % C: 10.3 %	2	Y^short^
	Kleinke et al. [68]	Germany	RCT	I: 85 C: 81	70.8 (4.8) I: 70.4 (4.6) C: 71.2 (5.0)	58.4 % I: 58.8 % C: 58.0 %	I: 16.7 % C: 22.1 %	3	N
	Lee et al. [69]	Taiwan	RCT	I: 102 C: 101	I: 71.3 (6.4) C: 71.3 (5.7)	I: 37.3 % C: 46.0 %	I: 10.8 % C: 7.0 %	2	Y^short^
	Mcmahon et al. [81]	USA	RCT	I: 16 C:14	83.6 (4.7)	93.3 %	7 %	2	Y^short^
	O'Brien et al. [70]	USA	RCT	I: 14 C: 10	69.0 (8.0)	100 %	I: 14.3 % C: 10.0 %	4	N
	Britten et al. [82]	UK	Mixed methods; Quasi-experimental	685	75.8 (9.4)	86 %	29.2 %	4	Y^short/long^
	Jang et al. [83]	Republic of Korea	Quasi-experimental	I: 11 C: 11	I: 72.5 (4.3) C: 68.6 (1.85)	36 % I: 45 % C: 27 %	9.1 %	2	Y^short^
	Jindo et al. [84]	Japan	Quasi-experimental	59	70.1 (3.5)	87 %	22 %	2	Y^short^
	Jones et al. [85]	USA	Quasi-experimental	237	71.5 (9.2)	81 %	68.8 %	3	Y^short/long^
	Joo et al. [86]	Korea	Quasi-experimental	14	69.5 (5.89)	0 %	0 %	3	Y^long^
	Kim et al. [87]	Korea	Quasi-experimental	I: 94 C: 130	I: 75.4 (5.9) C: 74.7 (5.7)	I: 94.7 % C: 88.5 %	NA	2	Y^long^
	Mcnamara et al. [88]	Australia	Mixed Methods; Quasi-experimental	7	NA	50 %	14.2 %	2	Y^short^
	Park et al. [89]	Republic of Korea	Quasi-experimental	46	73.41 (8.77)	82.6 %	0 %	2	Y^short^
	Paschoa and Ashton [90]	USA	Mixed Methods; Quasi-experimental	17	73	100 %	26 %	2	Y^short^
	Sowle et al. [91]	USA	Quasi-experimental	265	NA	83.4 %	42 %	2	Y^short^
	Strand et al. [92]	USA	Mixed Methods; Quasi-experimental	46	NA	87 %	32.4 %	3	Y^short^
	Yeh et al. [93]	Taiwan	Mixed Methods; Quasi-experimental	68	73.9 (9.1)	66.2 %	20 %	2	Y^short^
Poor diet (*N* = 6)	Goni et al. [73]	Spain	RCT	I: 365 C: 355	59.7 (10.7)	24 %	11 %	3	Y^long^
	Seangpraw et al. [74]	Thailand	RCT	I: 90 C: 85	NA	I: 53.3 % C: 57.6 %	0 %	3	Y^short^
	Al-Nimr et al. [72]	USA	Quasi-experimental	25	72.2 (5.8)	73.9 %	0 %	2	Y^short^
	Hamirudin et al. [94]	Australia	Quasi-experimental	79	85.5 (5.8)	52.9 %	13.90 %	2	Y^short^
	Sachdeva et al. [95]	India	Quasi-experimental	30	NA	0 %	NA	2	Y^short^
	Shahar et al. [96]	Malaysia	Quasi-experimental	I: 24 C: 23	I: 65.3 (3.5) C: 67.8 (4.7)	I: 63.6 % C: 60 %	I: 8.3 % C: 13 %	2	Y^short^
Social isolation (*N* = 9)	Li et al. [97]	China	RCT	I: 30 C: 30	I: 65.2 (2.6) C:= 65.6 (2.7)	I: 60.0 % C: 66.7 %	6.30 %	3	Y^long^
	Shapira et al. [98]	Israel	RCT	I: 64 C: 18	I: 72.1 (5.3) C: 71.7 (6.8)	I: 81 % C: 78 %	17 %	2	Y^short^
	Ashida et al. [99]	USA	Mixed Methods; Quasi-experimental	27	74.62 (NS)	81.5 %	10 %	2	Y^short^
	Banbury et al. [100]	Australia	Mixed Methods; Quasi-experimental	45	73 (7.2)	56 %	0 %	2	N
	Kikuchi et al. [101]	Japan	Mixed Methods; Quasi-experimental	I: 11 C: 6	I: 76.2 (6.9) C: 81.7 (3.1)	I: 63.6 % C: 36.4 %	0 %	2	Y^long^
	Kim et al. [102]	South Korea	Quasi-experimental	I: 39 C: 38	I:74.7 (5.3) C: 76.6 (5.5)	83.1 %	6.10 %	2	Y^short^
	Kim et al. [103]	South Korea	Quasi-experimental	10	82 (5.9)	100 %	37.50 %	2	Y^short^
	Oetzel et al. [104]	New Zealand	Mixed Methods; Quasi-experimental	I: 62 C: 54	I:= 68.2 (7.3) C: 71.5 (7.5)	I: 65.2 % C: 70.3 %	32.80 %	3	Y^long^
	Willard et al. [105]	Netherlands	Quasi-experimental	47	74 (6.2)	46.8 %	7.80 %	2	Y^short^
Depression (*N* = 12)	Brenes et al. [106]	USA	RCT	I: 70 C: 71	NA	I: 82.9 % C: 80.3 %	I: 25.0 % C: 19.0 %	3	Y^short^
	Chen et al. [107]	China	RCT	I: 1232 C: 1133	I: 74.4 (8.1) C: 4.6 (8.34)	I: 67 % C 66 %	I: 6.9 % C: 5.9 %	5	Y^short/long^
	Chojak [108]	Poland	RCT	I: 50 C: 50	75.6 (7.6)	80 %	I: 0 % C: 0 %	2	Y^short^
	Haringsma et al. [109]	Netherlands	RCT	I: 61 C: 58	64.2 (7.2)	69 %	I: 13 % C: 0 %	4	Y^short/long^
	Oh et al. [75]	South Korea	RCT	I: 82 C: 78	I: 74.9 (5.4) C: 73.5 (5.9)	I: 61.0 % C: 56.4 %	I: 0 % C: 21.8 %	2	Y^short^
	Scogin et al. [110]	USA	RCT	I: 70 C: 64	75.4 (7.1)	82.8 %	25 %	4	Y^short/long^
	Wang et al. [111]	China	RCT	I: 130 C: 160	I: 71.6 (7.7) C: 73.4 (6.9)	I: 63.6 % C: 63.5 %	I: 15.4 % C: 43.8 %	2	Y^short^
	Xie et al. [112]	China	RCT	I: 40 C: 40	I: 80.0 (4.0) C: 71.9 (3.7)	I: 57.5 % C: 60.0 %	I: 7.5 % C: 10 %	3	Y^short^
	Hollister et al. [113]	USA	Quasi-experimental	56	69.3 (7.4)	80.4 %	0 %	6	Y^short/long^
	Kim et al. [103]	South Korea	Quasi-experimental	10	82.0 (5.9)	100 %	37.50 %	2	Y^short^
	Sarkar et al. [114]	India	Quasi-experimental	263	NA	62.4 %	7.90 %	2	N
	Wu and Chao [115]	Taiwan	Mixed Methods; Quasi-experimental	29	NA	NA	25.60 %	2	Y^short^

RCT, Randomised Controlled Trial; I, Intervention group; C, Control group; NA, Not Available; Y, Intervention was effective (i.e. at least one outcome significantly improved at follow-up); N, Intervention was not effective (i.e. no outcomes significantly improved at follow-up); ^short^, significant short-term outcome (≤6 months); ^long^, significant long-term outcome (>6 months).

**Table 2:** Summary of intervention characteristics.

Risk factor	Author (year)	Description of intervention content	Control group	Intervention duration	Format		Setting	
					Group	Individual	At-home or distance delivered	Community or senior centre
Physical inactivity (N = 22)	Akihiroet al. [76]	Nurse-led exercise program	Usual care	3 months		X	X	
	Britten et al. [82]	Exercise dance program	–	12 months	X			X
	Cai et al. [77]	Psychologist and physical trainer-led physical activity group sessions, peer-led walking, and mobile application-based feedback and support	General health information	3 months	X	X		X
	Cruz-Ferreira et al. [78]	University dance teacher-led creative dance program	Usual care	24 weeks	X	X		X
	Harris et al. [71]	Individualised physical activity plan, pedometers behavioural strategies, physical activity handbook and diary provided	Usual care	3 months		X	X	
	Jang et al. [83]	mhealth app; wearable device; walking program; coaching by community health centre staff	–	13 months		X	X	
	Jeon et al. [79]	Research assistant/investigator-led exercise program	Usual care	12 weeks	X			X
	Jindo et al. [84]	Instructor led exercise program and recreational activities	–	11 weeks	X			X
	Johnson et al. [80]	Physiotherapist led exercise program, dietary advice, nutritional supplement, social support with a home support worker	Usual care	6 months		X	X	
	Jones et al. [85]	Trained instructor-led exercise program, DVD exercises	–	16 weeks	X			X
	Joo et al. [86]	Exercise program (stretching, strength training, aerobic exercise, new-Sports activity)	–	12 months	X			X
	Kim et al. [87]	Villager-led walking sessions; physical exercise specialists and community health practitioner-led gymnastics sessions	Usual care	7 months	X			X
	Kleinke et al. [68]	Researchers posted automatically generated physical activity monitoring and feedback letters	Usual care	3 months		X	X	
	Lee et al. [69]	Public heath nurse-led exercise program, individual contact via phone and face-to-face for motivation and reinforcing behaviours	Usual care	6 months		X	X	
	Mcmahon et al. [81]	Gerontological nurse practitioner-led exercise program; behavioural strategies; mHealth app	General health information	8 weeks	X	X	X	X
	Mcnamara et al. [88]	Exercise physiologist-led exercise program, cardiovascular and resistance exercise, balance and falls prevention	–	9 weeks	X			X
	O'Brien et al. [70]	Physical activity and diet intake monitoring and feedback with peer support	General health information	12 weeks		X	X	
	Park et al. [89]	Weekly instructor led exercise program	–	8 weeks	X			X
	Paschoa and Ashton [90]	Exercise program; behavioural strategies	–	6 weeks	X			X
	Sowle et al. [91]	Youth-trainer led exergaming and interactive games, newsletter	–	8 weeks	X	X	X	X
	Strand et al. [92]	Youth-trainer-led exergaming, newsletter, cookbook, physical activity guidelines and instructional DVD	–	25 weeks	X	X	X	X
	Yeh et al. [93]	Nurse-led exercise program; recreational activities, interactive entertainment games	–	12 weeks	X			X
Poor diet (*N* = 6)	Al-Nimr et al. [72]	Dietitian-led counselling, physical therapist-led exercise program	–	12 weeks	X	X	X	X
	Goni et al. [73]	Phone contact with dietitian; web-based intervention on Mediterranean diet, dietary recommendations, web page, mobile app, and printed resources	Usual care	2 years		X	X	X
	Hamirudin et al. [94]	Dietician delivered individualised clinical dietary advice	–	12 weeks		X	X	
	Sachdeva et al. [95]	Nutritional counselling via lectures, discussions, demonstrations and visual aids	–	3 months		X	NA	NA
	Seangpraw et al. [74]	Guidebook for behaviour modification guidelines a dietician-led diet program with self-efficacy, group education meetings, group activity training sessions, and an individual checklist	Usual care	12 weeks	X	X	X	X
	Shahar et al. [96]	Dietitian-led group counselling sessions with nutrition education, talks, cooking classes, exercise demonstrations and resources	–	6 months	X	X		X
Social isolation (*N* = 9)	Ashida et al. [99]	PrepWise program, disaster preparedness training program led by an experienced disaster preparedness educator	–	1 month	X			X
	Banbury et al. [100]	Telehealth literacy project led by experienced health promotion professional	–	6 weeks	X		X	
	Kikuchi et al. [101]	Social Activity Program that Encourages Interaction (SAPEI)-Facilitator led face-to-face activities at the senior citizen club; communication app (Kikoeru) and pedometer	–	13 months	X	X	X	X
	Kim et al. [102]	Integrated healthcare program led by researcher	Usual care	12 weeks	X			X
	Kim et al. [103]	Multi-component program (3 parts) led by National Social Prescribing Network, delivered by experts, participants and university student volunteers. Involved music storytelling, a self-help group and gardening.	–	10 weeks	X			X
	Li et al. [97]	Chinese traditional festival activities-Group Reminiscence Therapy (CTFA-GRT) program led by team of healthcare experts	Usual care	8 months	X			X
	Oetzel et al. [104]	Tuakana-teina/peer education orientation programme for life-transitions of kaumātua-peer educators (tuakana)	–	12–16 weeks	X	X		X
	Shapira et al. [98]	Online guided group sessions led by a clinical social worker. Involved learning cognitive and behavioural techniques	Usual care	4 weeks	X		X	
	Willard et al. [105]	Online Community Care Platform; calendar for local activities and events, service wayfinding	–	4 months		X	X	X
Depression (*N* = 12)	Brenes et al. [106]	Clinical psychologist and social workers delivered telephone Cognitive Behavioural Therapy sessions	Non-directive support therapy (NST-T)*	4 months		X	X	
	Chen et al. [107]	Chinese Older Adult Collaborations in Health (COACH) integrated care intervention; primary care provider (screening), ageing worker (education, goal setting, behaviour change strategies) and hospital psychiatrist (medication administration and review).	Enhanced Care-as-Usual†	12 months		X	X	
	Chojak [108]	Psychologist or social therapist led Acceptance and Commitment Therapy (ACT) program.	Positive Psychology Intervention (PPI) ‡	12 weeks	X			X
	Haringsma et al. [109]	Health care professional led Coping with Depression course (CWD)	Usual care	10 weeks	X			X
	Hollister et al. [113]	Clinician led Case Management with Problem Solving Therapy (CM-PST)	–	12 weeks		X	X	
	Kim et al. [103]	Multi-component social prescribing program delivered by experts, participants and university student volunteers. Involved music storytelling, a self-help group and gardening.	–	10 weeks	X			X
	Oh et al. [75]	Community Mental Health Staff (CMHS) led individual case management community visits and group-based intervention	Usual care	12 weeks	X	X	X	X
	Sarkar et al. [114]	Problem solving therapy (PST) and Brief Intervention (BI) counselling	–	12 months	X	X	X	X
	Scogin et al. [110]	Cognitive Behavioural Therapy led by students or clinical social worker	Minimal support§	12 weeks	X	X	X	
	Wang et al. [111]	Solution-Focused Group Counselling (SFGC) led by local healthcare worker-led	Usual care	6 months	X			X
	Wu and Chao [115]	Beauty program led by experienced beauty training instructor and collaboratively developed and informed by beautician, registered nurse, occupational therapist and reviewed by health promotion expert	–	15 weeks	X			X
	Xie et al. [112]	Facilitator (post graduate nursing student) led Modified Behavioural Activation Treatment (MBAT)	Usual care	8 weeks	X			X

NA, not available.

⁎ Supportive and reflective communications only, no advice, suggestions or coping methods.

† Provided anti-depressant treatment guidelines when patients screened for depression.

‡ Practicing gratitude, building awareness of the most positive aspects of oneself, and identifying strengths of character and relaxation training.

§ No Cognitive Behavioural Therapy; brief weekly telephone calls from project staff, monitoring of participants.

For physical inactivity, there was a total of 2720 participants, mean age ranged from 67 to 84, proportion of females ranged from 0 to 100 %, with a total of 22 interventions implemented across these studies ranging from 6 weeks to 12 months in duration. Most were conducted in a group setting (*N* = 15/22) and in-person at a community or recreation centre (*N* = 15/22). Interventions were predominantly educational and psychosocial, incorporating a structured program of resistance and aerobic-based training in which participants were guided or instructed by a health professional (e.g., public health/gerontological nurse) or a trainer (*N* = 15/22). Other interventions were creative and recreational-based (e.g., creative dance, exergaming) (*N* = 5/22), app-based (e.g., Fitbit) (*N* = 5/22), and peer and youth trainer-led (*N* = 3/22). 8 interventions touched on topics in addition to physical activity such as nutrition, wellbeing, mental health, falls prevention, peer support and motivation. There were 54 primary outcomes and 35 secondary outcomes across studies with majority of studies measuring short-term (≤6 months) (*N* = 21/22) compared to long-term outcomes (>6 months) (*N* = 6/22).

For poor diet, there was a total of 1076 participants, mean age ranged from 60 to 86 and proportion of females ranged from 0 to 74 %. Six interventions were identified, and more than half were 12 weeks in duration (4/6 studies, range = 12 weeks–2 years). Almost all interventions were delivered or informed by a registered dietitian or nutritionist (*N* = 5/6), were delivered in a group and/or individual setting (*N* = 4/6) with majority offering individual or at-home program options (*N* = 4/6). Most interventions (*N* = 4/6) involved nutritional counselling and provided participants with additional resources in the form of online materials (webpage or app), printed resources (flipchart, workbook, diet checklist) or physical resources (olive oil, meals on wheels, cooking demonstrations and placemats). A total of 14 primary outcomes and 2 secondary outcomes, with 5 studies that measured outcomes in the short term only (≤ 6 months), and 1 study in the long-term (> 6 months) were found.

For social isolation, there was a total of 486 participants, mean age ranged from 66 to 82, proportion of females ranged from 47 to 100 %, most were conducted in group settings (*N* = 6/9) at a community or senior centres (*N* = 4/9) and adopted both online and in-person formats (*N* = 4/9). Whilst all focused on social support promotion, other elements included an emphasis on local context and cultural influences (*N* = 6/9), as well as practical application and conversation (*N* = 5/9), whilst others were predominantly informational or instructional, incorporating lectures and modules (*N* = 3/9). Interventions primarily ran from 4 weeks to 13 months, measured 26 primary outcomes and no secondary outcomes typically at short-term follow up time points (≤ 6 months) (*N* = 7/9) compared to long-term observations (> 6 months) (*N* = 3/9) with 1 study measuring outcomes at short and long-term time points.

For depression, there was a total of 3747 participants, mean age and proportion of females ranging from 64 to 82 and 56–100 %, respectively. Most interventions used Cognitive Behavioural Therapy (*N* = 7/12), and interventions included activities that provided psychosocial support (e.g., self-help groups, case management and connection to community services, music storytelling, gardening, and beauty program for perception of ageing) (*N* = 9/12). Almost all interventions were delivered by a professional (e.g., psychologist, social worker, primary health care worker) (*N* = 10/12) whilst the remainder of studies enlisted assistance from university students to deliver the interventions (*N* = 2/12). Intervention duration was 8 weeks through to 12 months and most were conducted in-person in a group setting at a community or recreation centre (*N* = 6/12). In contrast, majority of the individual intervention formats (*N* = 2/3) were delivered over-the-phone. There were 26 primary outcomes and 17 secondary outcomes, with most measuring short-term outcomes (≤ 6 months) (*N* = 11/12) compared to long-term outcomes (*N* = 5/12) (> 6 months), and 3/12 studies included assessments at both timepoints.

### 3.2 BCT categories

**Fig. 3:**
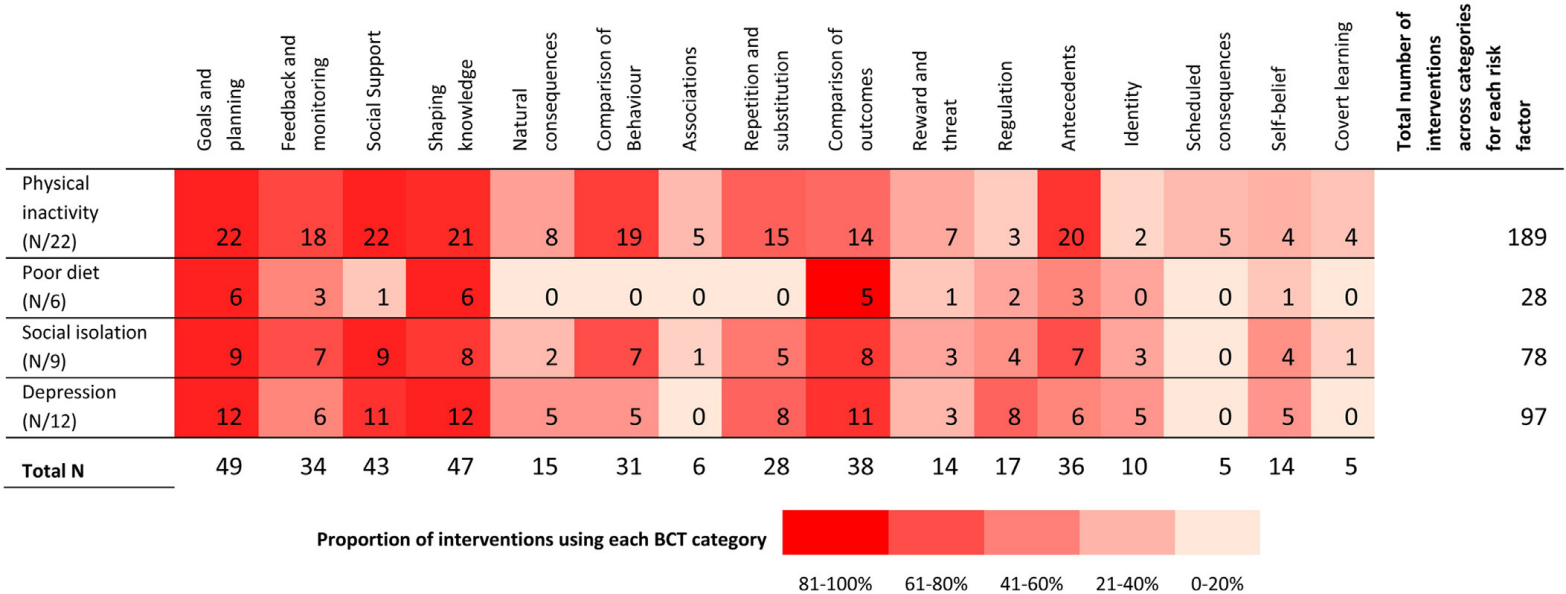
Number and proportion of interventions that used each BCT category according to each risk factor.

There was an average of 14.8 BCTs (calculated out of the 93 BCTs) present (SD = 10.0) per study, which captured all 16 BCT categories. Fig. 3 presents the proportion of interventions that used each BCT category according to each risk factor. Almost a quarter of (21/93; 22.6 %) BCTs were not present in any of the interventions. The most commonly occurring BCT categories across risk factors included goals and planning (49/49 interventions; 100 %), shaping knowledge (47/49 interventions; 95.9 %), social support (43/49 interventions; 87.8 %) and comparison of outcomes (38/49 interventions; 77.6 %).

### 3.3 BCTs

**Fig. 4:**
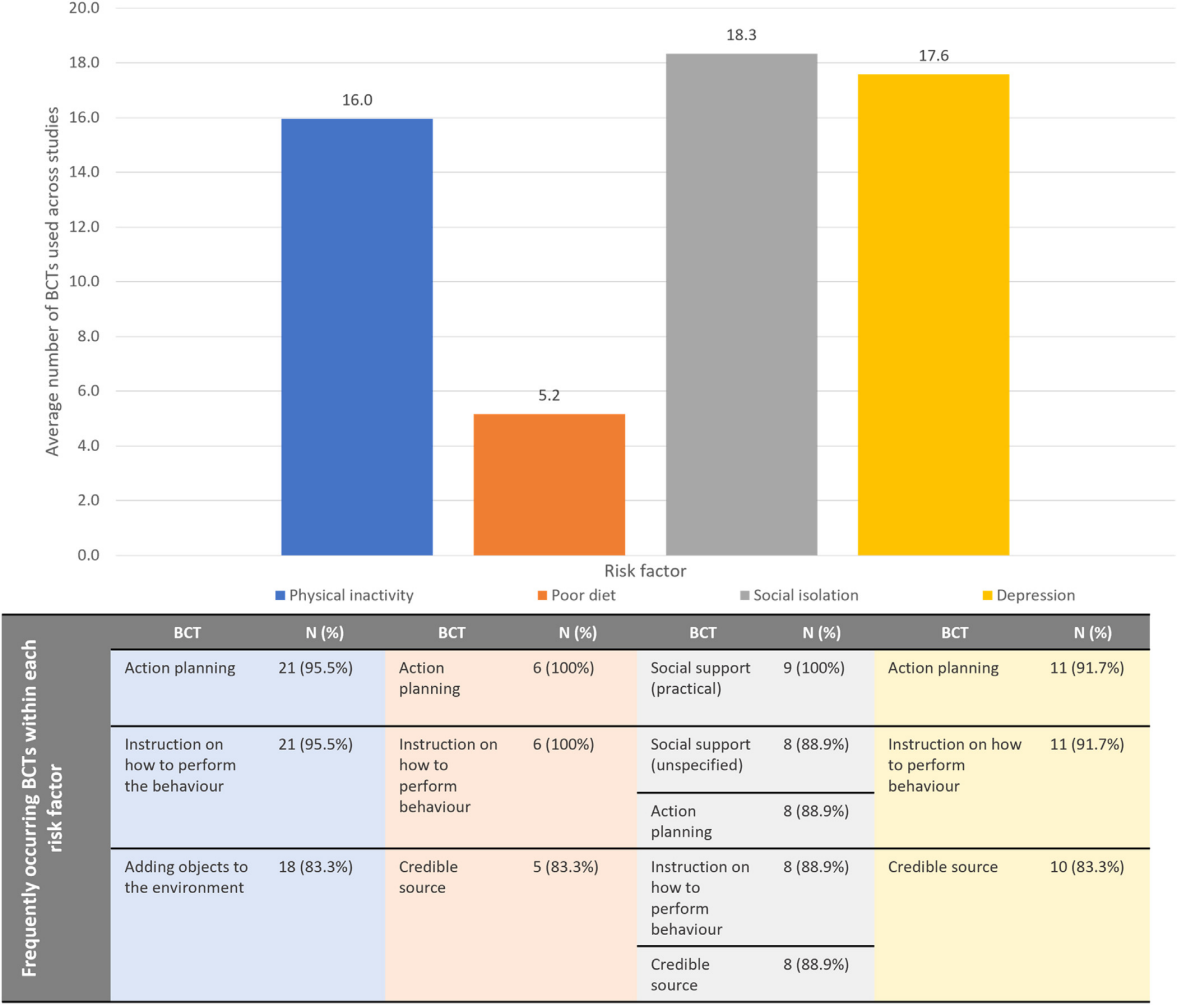
Average number and frequently occurring BCTs used within each risk factor.

Out of the 93 BCTs, the most commonly applied BCTs were “instruction on how to perform the behaviour” (47/93; 50.5 %), “action planning” (46/93; 49.4 %) and “credible source” (38/93; 40.9 %). Physical inactivity interventions had the most BCT categories coded with a total of 67/93 (72.0 %) BCTs adopted, followed by social isolation (54/93; 58.0 %), depression (49/93; 52.7 %) and diet (12/93; 12.9 %) interventions. Fig. 4 demonstrates the average number of BCTs coded across interventions for physical inactivity, poor diet, social isolation and depression as well as the most commonly used BCTs across studies. Programs reducing social isolation had the highest average of BCTs applied in their interventions (M = 18.3; SD = 8.5) followed by interventions targeting depression (M = 17.6; SD = 10.7), physical inactivity (M = 16.0; SD = 11.5) and poor diet (M = 5.2; SD = 3.0). Across all risk factors, most commonly used BCTs included “action planning”, “instruction on how to perform the behaviour” and “credible source”.

### 3.4 BCTs and effectiveness

Overall, across all interventions, there were 4 studies out of the total 49 studies that reported no significant change in outcomes as a result of their interventions; 2 targeting physical inactivity [68,70], 1 for social isolation [100] and 1 addressing depression [114]. There were few obvious differences in BCTs identified in studies considered not effective compared to those that did report significant outcomes. However, 3 out of 4 studies [68,100,114] had fewer than the average number of BCTs applied compared to the effective interventions for each risk factor. We then examined patterns for each risk domain separately.

Two ineffective physical inactivity interventions [68,70] lacked key behaviour change techniques. Both were missing the commonly used “demonstration of behaviour”, and Kleinke's intervention did not include “adding objects to the environment” [68]. O'Brien's study, in contrast, had a higher use of techniques in the reward and threat category compared to similar, effective interventions [70].

All interventions targeting diet had at least one significant outcome, thus no patterns were identified in frequency or type of BCT use and effectiveness.

As for reducing social isolation, there was no identifiable difference between BCTs applied in the single ineffective intervention [100] compared to effective interventions. However, when considering the intervention characteristics in Table 2, Banbury and colleagues’ [100] intervention was 1 of only 2 studies to deliver an online only intervention without face-to-face interaction. Intervention content heavily differed between these 2 online studies.

Within interventions targeting depression, the single ineffective study [114] was the only one that did not apply the “action planning” BCT, while other common BCTs like “instruction on how to perform the behaviour” and “credible source” showed no differences. Sarkar and colleague's intervention [114] focused on group social activities, including health education, games, and TV viewing, unlike effective interventions that typically used Cognitive Behavioural Therapy (e.g., [[106], [107], [108], [109],^111^,^112^] and was only offered to select participants, with low completion rates.

### 3.5 Risk of bias

Across 15 available RCT studies, risk of bias was assessed as low with some studies reporting some concerns. Main concerns were primarily related to the nature of lifestyle interventions, where controlling for allocation concealment and performance bias was challenging. However, these studies either addressed baseline differences in their analysis plan or had clear protocols for intervention delivery. Three studies were assessed as high risk due to blinding outcome assessment bias. Akihiro and colleagues [76] had the highest risk of bias which was a result of a randomisation difficulties due to recruitment issues, control group exposure to an intervention of a similar nature (i.e., a nurse-led community exercise class), and a lack of assessor blinding.

### 3.6 Meta-analysis

**Fig. 5:**
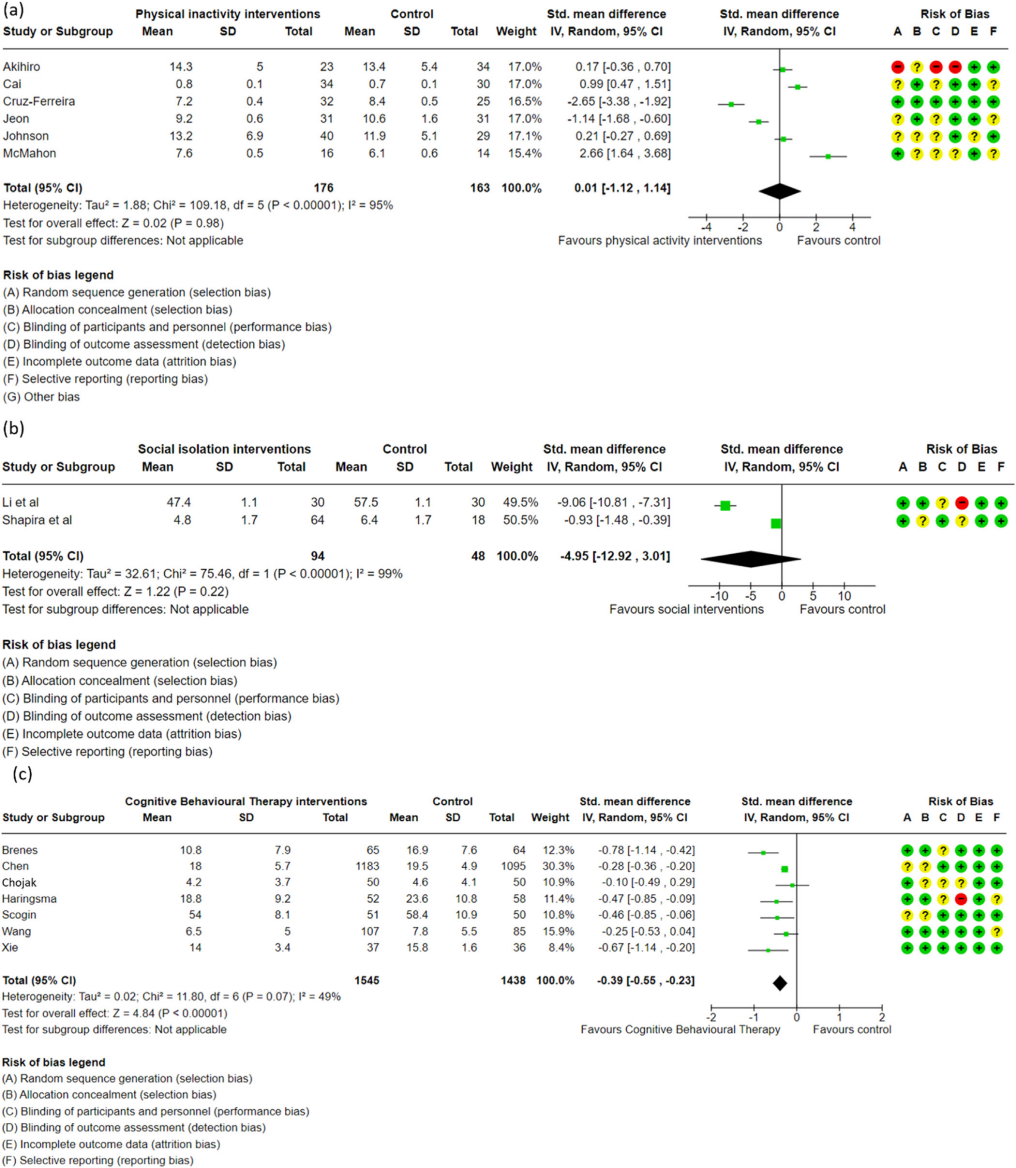
Meta-analysis, a) Physical inactivity interventions, b) Social isolation interventions, c) Cognitive Behavioural Therapy interventions.

The meta-analysis of 6 RCTs [[76], [77], [78], [79], [80], [81]] indicated that physical inactivity-based interventions for adults over 55 in rural areas may have little to no effect on physical function compared to usual care, but based on the GRADE criteria [62], the evidence was highly uncertain (SMD 0.01, 95 % CI −1.12 to 1.14) (Fig. 5a). For social isolation (Fig. 5b) the meta-analysis of 2 RCTs [97,98] showed low-certainty evidence that behavioural interventions may result in little to no difference in loneliness (SMD −4.95, 95 % CI −12.92 to 3.01). Regarding depression (Fig. 5c), the meta-analysis of 7 RCTs [[106], [107], [108], [109], [110], [111], [112]] provided moderate-certainty evidence that Cognitive Behavioural Therapy likely reduces depressive symptoms in this population (SMD −0.39, 95 % CI −0.55 to −0.24). Significant heterogeneity was observed in the meta-analyses for physical inactivity and social isolation, suggesting potential variability in some studies and caution is advised in interpreting these results.

## 4 Discussion

This review identified BCTs within interventions targeting modifiable dementia risk factors independently in rural and regional older adults. We found a limited number of empirical behavioural interventions focusing on improving physical inactivity, poor diet, social isolation and depression for this population. Most interventions were short-term (≤6 months), and few measured long-term outcomes (>6 months). Our synthesis identified shared BCT categories such as goals and planning (49/49 interventions; 100 %), shaping knowledge (47/49 interventions; 95.9 %), social support (43/49 interventions; 87.8 %) and comparison of outcomes (38/49 interventions; 77.6 %) as well as commonly applied BCTs such as “instruction on how to perform the behaviour”, “action planning” and “credible source”, across the 49 interventions. Inconsistent outcome measures across studies limited the meta-analyses, which indicated that physical inactivity interventions had minimal impact on physical function (very low-certainty), social isolation interventions had little to no effect on loneliness (low-certainty) and interventions incorporating Cognitive Behavioural Therapy for depression significantly improved depressive symptoms (moderate-certainty).

### 4.1 Physical inactivity interventions

Our finding that behavioural interventions targeting physical function were not effective is unexpected given previous research findings from the general adult population [50,51,116]. The commonly used BCTs identified in our review, such as “action planning”, “demonstration of behaviour”, and “adding objects to the environment”, aligned with those proven effective in prior physical inactivity interventions for adults [116]. A recent systematic review further supports these specific techniques for improving diet and physical activity in the long term among individuals with chronic conditions [117], highlighting their role in enabling self-sustaining change and habit formation alongside “prompts and cues,” “goal setting” and “self-monitoring” [118]. Despite this alignment, the studies in our review did not yield effective outcomes in the meta-analysis. This discrepancy may stem from several factors. First, many studies had small sample sizes and employed inconsistent outcome measures, complicating comparative analysis (e.g., step count, gait speed). Second, the populations studied were relatively healthy and physically active at baseline, which could attenuate the observed effects of the interventions. Third, most programs had short durations; 19 out of 24 interventions lasted less than six months. While shorter programs may initiate behaviour change, they are insufficient for maintenance and achieving significant changes in objective outcomes such as anthropometric measures (e.g., BMI, waist-to-hip ratio) and physical function (e.g., Timed Up and Go) [118]. Additionally, certain combinations of BCTs and intervention components have been found to influence physical activity which we were not able to investigate in our meta-analysis. For example, “action planning” with “self-monitoring” [50] compared to “action planning” alone, reduced exercise levels, and interventions that were person-centred and focused on building autonomy have been associated with behaviour maintenance [51].

### 4.2 Poor diet interventions

In the narrative synthesis, behavioural interventions targeting diet in rural communities demonstrated significant improvements in anthropometric outcomes including reductions in weight, waist circumference, blood glucose and cholesterol levels. However, a meta-analysis was not feasible due to substantial variation in measures and outcomes, coupled with limited literature on dietary behaviours in rural areas [119]. In contrast to physical inactivity interventions, which often require longer durations to show significant effects, dietary interventions in our review demonstrated significant impacts on objective measures despite shorter intervention periods (3 months in 63 % of the programs). This finding suggests the potential efficacy of dietary changes for rural older adults compared to having the dominant component of interventions focused on physical inactivity [120]. Previous reviews have frequently explored the impact of BCTs in interventions targeting both diet and physical activity simultaneously [51,121,122] with one review reporting a combination of BCTs being twice as effective at addressing physical activity and healthy eating than the studies that did not do this [122]. There is however, very limited research that examines diet and exercise interventions separately in rural settings, perhaps reflecting the perception of a higher likelihood of achieving positive outcomes when modifying both diet and physical activity behaviours concurrently [120]. However, this could mean BCTs that are specifically effective for changing diet as opposed to physical activity may have been overlooked. In our review, physical inactivity interventions tended to use more BCTs than poor diet interventions (M = 16.0 vs. M = 5.2) and apart from “action planning” the categories and types of BCTs used were inconsistent. Samdal and colleagues highlight that BCTs which tap into self-regulation explain intervention effects for both types of interventions especially in long-term. Future trials of exercise and nutrition interventions in rural and regional areas could adopt BCTs unique to each risk factor that can facilitate self-regulation and motivation to increase the likelihood of intervention effectiveness.

### 4.3 Social isolation interventions

To our knowledge, there was no existing review exploring BCTs within behavioural interventions that target social participation, social isolation or loneliness for rural and regional older adults. This was surprising given that understanding how to form and maintain a strong social fabric to reduce loneliness is valuable in a post COVID-19 pandemic context, especially amongst older adults who continue to be at higher risk of social isolation [123]. However, the lack of studies exploring underlying mechanisms may be due to the difficulty in delineating outcomes commonly measured in social isolation interventions with mental health and wellbeing outcomes (i.e., loneliness, self-efficacy, life satisfaction, depressive symptoms, stress). The present meta-analysis of behavioural interventions for social isolation indicated no evidence for impact on loneliness. This is inconsistent with a recent review on interventions targeting social isolation and loneliness amongst older metropolitan-based adults which despite high between-study heterogeneity, found a medium effect size overall and larger effect sizes for non-technology interventions [124].

Much of the behaviour change literature on social isolation is rooted in social prescribing, a growing initiative that links individuals to community-based opportunities aimed at enhancing health and wellbeing, particularly in offsetting loneliness and supporting pandemic recovery [125]. Key BCTs in effective social prescribing interventions include “social support (unspecified)”, “credible source” and “social support (practical)” [126]. While only one study in rural and regional adults used social prescribing [103], the most frequently adopted BCTs and categories for social isolation in our review (i.e. “social support”, “goals and planning” and “comparison of outcomes”) are not fully aligned with creating grass-root opportunities, as is common in social prescribing interventions [126].

### 4.4 Depression interventions

Behavioural interventions targeting depression provided the most certainty of evidence across risk factors. This result is likely due to the incorporation of Cognitive Behavioural Therapy methods (e.g. Modified Behavioural Activation treatment (MBAT) [112], Acceptance and Commitment Therapy [108], Coping with Depression (CWD) course [109]). Rooted in cognitive theory [127,128], Cognitive Behavioural Therapy is structured to identify maladaptive thought patterns and teach practical skills for everyday applications. It has shown to be effective in treating psychological conditions like depression, anxiety, and mood and personality disorders as well as managing chronic conditions, pain, and fatigue [129].

In our review, “action planning” and “instruction on how to perform the behaviour” were frequently identified and have demonstrated effectiveness within the broader literature. Specifically “instruction on how to perform the behaviour” has promoted health in older migrants [54] and improved quality of life in older adults when combined with “goal setting”, “problem solving” and “social support” [130]. Interestingly, the one intervention that failed to improve depression outcomes adopted “problem solving” and “social support”, but lacked “action planning” [114], which has been incorporated into primary care for almost 2 decades and has been shown to increase the likelihood of behaviour change when planning details such as setting, frequency, and intensity are considered [131]. It has also been successfully applied to multiple behaviours that contribute to improvements in depression outcomes such as exercise and diet [50,116]. Implications of our findings suggest that future research should investigate which combinations of BCTs that make-up Cognitive Behavioural Therapy (i.e., those that address cognitive, emotional and affective process in a structured manner) are most effective in transforming intentions into practical steps not only to achieve improvements in depressive outcomes but general health behaviours and dementia risk as an outcome.

### 4.5 BCTs and MoAs and risk factors for dementia

BCTs enable behaviour change either by increasing factors that facilitate wanted behaviour or inhibit unwanted behaviour. Behaviour change theory suggests that selecting BCTs based on the types of implicit processes within individuals and groups through which they have their effects, notably Mechanisms of Action (MoAs) will lead to a better understanding of how and why the intervention may be effective or not [132,133]. For example, if the MoA to target is subjective norms, BCTs “social comparison” and “information about other's approval” are strongly linked to changing an individual's perception on norms [134]; if the MoA is environmental context and resources, adopting the BCTs “prompts/cues”, “adding objects to the environment” or “restructuring the physical environment” are best suited [133].

Across all interventions in this review, despite whether it was effective or not, “action planning” was the most frequently occurring BCT, followed by “instruction on how to perform the behaviour” and “credible source”. Within the published intervention literature, these BCTs most frequently occur with MoAs behavioural regulation (having the behaviour, cognitive and emotional skills to change behaviour), knowledge (awareness that the behaviour exists), and general attitudes and beliefs (the evaluation of that person, group or issue on a positive to negative scale), which could suggest that similar MoAs may be present across these 4 dementia risk factors [132]. However, according to an expert consensus study, the BCTs “action planning” and “credible source” may not be the most effective tools for targeting these MoAs since no definitive links to behaviour change could be identified [133]. Rather, MoA behavioural regulation was linked to BCTs “problem solving” and “reducing negative emotions”, whereas there were no BCTs directly linked with general attitudes and beliefs MoA [133]. Therefore, in order to facilitate behaviour change, irrespective of which risk factor or health behaviour is being targeted (e.g., in multidomain interventions), understanding the MoA required to initiate and sustain behaviour change and adopting appropriate BCTs may produce better outcomes.

Further research should seek to establish whether these MoAs differ according to contextual or socio-environmental factors (i.e., socioeconomic status [135], ethnicity, remoteness and population density) which may influence intervention design to address existing health disparities among disadvantaged populations (e.g., food insecurity and diet quality in rural and regional older adults [136]). For example, in our meta-analyses, social isolation interventions were not effective at reducing loneliness. BCTs involved in social prescribing may be a favourable course of action for adults indicating they are lonely, however loneliness is also linked to neighbourhood disadvantage, reduced access to services, including transport, as well as open green spaces [137]. Similarly, physical inactivity interventions did not improve physical function and previous research indicates distance, reduced walkability and facility access, and neighbourhood safety concerns are key barriers to reducing physical inactivity. Therefore, an alternative intervention pathway to address social isolation and physical inactivity may be through the MoA, environmental context and resources. Operationalising this involves Regulation BCTs such as “restructuring the physical environment”, and “avoiding/reducing exposure to cues for the non-desirable behaviour”. In a rural-specific context this may involve zoning or re-allocating road space for pedestrians, town planning for 15-minute neighbourhoods, improving access to communication technology and internet facilities, or investment in better transport services [138,139]. Whilst these types of interventions using BCTs targeted at MoAs on a socio-environmental level may be a useful step in reducing dementia risk factors in rural areas, more documented evidence of effectiveness in rural communities is firstly necessary as it involves significant political, financial and human capital commitment [140]. Simultaneously, BCTs linked with individualised MoAs (e.g., BCT “behavioural practice/rehearsal” and MoA, beliefs about capabilities [132]) should not be ignored in future interventions for dementia risk, especially when targeting high-risk populations.

### 4.6 Strengths and limitations

To our knowledge, this is the first review to identify interventions targeting single risk factors for dementia amongst rural and remote older adults. The small number of studies reiterates the differences in access to these types of behavioural interventions for improving lifestyle amongst regional populations. The extrapolation and synthesis of BCTs across risk factors can be used as a theoretical guide to improve future intervention design and delivery as well as encourage standardised reporting of BCTs for replication in the literature.

However, there are several limitations. Firstly, there were only a small number of comparison studies for each risk factor meta-analyses, most of which had small sample sizes (1/15 studies >1000 participants) and very low to low certainty of evidence (2/3 outcomes). While a strength of this study is extrapolating BCTs targeting single risk factors independently to identify what is commonly used and which BCTs may work better than others for specific risk factors, more studies could have been included if certain combinations of risk factors (i.e., physical activity and depression) or other outcomes affiliated with dementia (e.g., cognition) were investigated. Second, due to the small number of studies it was only possible to conduct a narrative synthesis of BCTs and direct associations with individual BCTs or clusters of BCTs were not examined. Third, coding the BCTs was difficult at times as coding assumptions were restricted to explicit details. Only one intervention referenced the BCT taxonomy directly which is a commonly cited limitation in intervention implementation [141]. Where referenced, other studies and websites were consulted to obtain further details. Adoption and direct signposting of the BCT taxonomy is recommended in the design and implementation of future interventions [49].

The limited effectiveness of interventions may also be attributed to their poor applicability within rural and regional areas. Traditionally, a “one size fits all” approach has been applied to all regional areas but variations in community characteristics creates unique barriers to implementing physical inactivity, poor diet, social isolation and depression interventions [28,142]. These include individual level preferences (e.g., older adults are less likely to desire change because their behavioural patterns are more ingrained, they receive less support from family and friends and are not exposed to alternative social norms because of their isolation [143,144]), geographic barriers (e.g., transportation availability and costs, safety concerns, internet and technology accessibility, neighbourhood walkability, distance from large-chain supermarkets [143,144]), incompatibility with existing familiar schedules (e.g., loyalty to local grocers), and lack of infrastructure (limited healthcare staff, allied health, specialists and facilities) to support program integration into the community. Only 7/47 studies assessed feasibility and acceptability [81,83,85,100,102,104,105], whilst some others briefly mentioned program satisfaction, engagement (e.g., [80,115]), and strategies to produce higher retention rates in rural areas [106]. Future studies could seek to create locally relevant and sustainable interventions by forming community connections and involvement early on and implementing BCTs known to enhance retention and long-term engagement in programs targeting risk factors for dementia (e.g., antecedents, and reward and threat BCT categories for diet and physical activity interventions [145,146]).

## 5 Conclusion

The current review and meta-analysis identified BCTs within interventions targeting four single modifiable risk factors for dementia including physical inactivity, poor diet, social isolation and depression. Consistencies amongst BCT categories and the frequent use of BCTs “action planning” and “instruction on how to perform the behaviour” align with previous literature and could indicate their usefulness in interventions targeting single or multiple dementia risk factors among rural and regional adults. However, the meta-analyses were limited and conclusions about effectiveness of interventions are weak due to the small number of studies, limited power, and insufficient exploration of longer-term outcomes. This suggests that further investigation of BCTs that influence feasibility and acceptability outcomes, in addition to tailoring intervention design with and for each regional community, may be useful. By considering the socio-environmental context, such tailored approaches could enable greater engagement, sustainability and impact in reducing dementia risk.

## Funding

This research is funded by Western Sydney University Research Training Program (RTP) Postgraduate Research Scholarship Award to LD.

## CRediT authorship contribution statement

**Laura Dodds:** Writing – review & editing, Writing – original draft, Visualization, Validation, Investigation, Formal analysis, Data curation, Conceptualization. **Kay Deckers:** Writing – review & editing, Validation, Supervision. **Celia B. Harris:** Writing – review & editing, Validation, Supervision. **Joyce Siette:** Writing – review & editing, Writing – original draft, Visualization, Validation, Supervision, Software, Resources, Methodology, Formal analysis, Data curation, Conceptualization.

## Declaration of competing interest

The authors declare that they have no known competing financial interests or personal relationships that could have appeared to influence the work reported in this paper.

## Data Availability

Data will be made available upon request to the author.

## Appendix Supplementary materials

Image, application 1
