# Supplementary material for: Behaviour change techniques used in interventions targeting dementia risk factors amongst older adults in rural and remote areas: A systematic review and meta-analysis
Source: J Prev Alzheimers Dis. 2025 Feb 22;12(4):100093. doi: 10.1016/j.tjpad.2025.100093 (PMC12184004; doi:10.1016/j.tjpad.2025.100093)
Supplement: Supplementary file 1 [file mmc1.docx]

**Appendices**

**Appendix I. Link to full list of BCTs, BCT definitions, and examples**

Reproduced from S. Michie, M. Richardson, M. Johnston, C. Abraham, J. Francis, W. Hardeman, et al. Ann Behav Med 2013 Vol. 46 Issue 1 Pages 81-95. DOI: 10.1007/s12160-013-9486-6. Access via [<https://digitalwellbeing.org/wp-content/uploads/2016/11/BCTTv1_PDF_version.pdf>]

**Appendix II. PRISMA checklist**

| 1. **Section and Topic** | **Item #** | **Checklist item** | **Location where item is reported** |
| --- | --- | --- | --- |
| **TITLE** | | |  |
| Title | 1 | Identify the report as a systematic review. | Pg 1 |
| **ABSTRACT** | | |  |
| Abstract | 2 | See the PRISMA 2020 for Abstracts checklist. | Pg 2 |
| **INTRODUCTION** | | |  |
| Rationale | 3 | Describe the rationale for the review in the context of existing knowledge. | Pg 3-4 |
| Objectives | 4 | Provide an explicit statement of the objective(s) or question(s) the review addresses. | Pg 4 |
| **METHODS** | | |  |
| Eligibility criteria | 5 | Specify the inclusion and exclusion criteria for the review and how studies were grouped for the syntheses. | Pg 6 |
| Information sources | 6 | Specify all databases, registers, websites, organisations, reference lists and other sources searched or consulted to identify studies. Specify the date when each source was last searched or consulted. | Pg 6-7 |
| Search strategy | 7 | Present the full search strategies for all databases, registers and websites, including any filters and limits used. | Appendix II |
| Selection process | 8 | Specify the methods used to decide whether a study met the inclusion criteria of the review, including how many reviewers screened each record and each report retrieved, whether they worked independently, and if applicable, details of automation tools used in the process. | Pg 6-7 |
| Data collection process | 9 | Specify the methods used to collect data from reports, including how many reviewers collected data from each report, whether they worked independently, any processes for obtaining or confirming data from study investigators, and if applicable, details of automation tools used in the process. | Pg 6-7 |
| Data items | 10a | List and define all outcomes for which data were sought. Specify whether all results that were compatible with each outcome domain in each study were sought (e.g. for all measures, time points, analyses), and if not, the methods used to decide which results to collect. | Pg 6-7 |
|  | 10b | List and define all other variables for which data were sought (e.g. participant and intervention characteristics, funding sources). Describe any assumptions made about any missing or unclear information. | Pg 6-7 |
| Study risk of bias assessment | 11 | Specify the methods used to assess risk of bias in the included studies, including details of the tool(s) used, how many reviewers assessed each study and whether they worked independently, and if applicable, details of automation tools used in the process. | Pg 8 |
| Effect measures | 12 | Specify for each outcome the effect measure(s) (e.g. risk ratio, mean difference) used in the synthesis or presentation of results. | Table 1, Table 2, Figure 5 |
| Synthesis methods | 13a | Describe the processes used to decide which studies were eligible for each synthesis (e.g. tabulating the study intervention characteristics and comparing against the planned groups for each synthesis (item #5)). | Pg 6-7 |
|  | 13b | Describe any methods required to prepare the data for presentation or synthesis, such as handling of missing summary statistics, or data conversions. | Pg 6-8 |
|  | 13c | Describe any methods used to tabulate or visually display results of individual studies and syntheses. | Pg 6-8 |
|  | 13d | Describe any methods used to synthesize results and provide a rationale for the choice(s). If meta-analysis was performed, describe the model(s), method(s) to identify the presence and extent of statistical heterogeneity, and software package(s) used. | Pg 8 |
|  | 13e | Describe any methods used to explore possible causes of heterogeneity among study results (e.g. subgroup analysis, meta-regression). | Pg 8 |
|  | 13f | Describe any sensitivity analyses conducted to assess robustness of the synthesized results. | Pg 8 |
| Reporting bias assessment | 14 | Describe any methods used to assess risk of bias due to missing results in a synthesis (arising from reporting biases). | Pg 8 |
| Certainty assessment | 15 | Describe any methods used to assess certainty (or confidence) in the body of evidence for an outcome. | Pg 8 Appendix III |
| **RESULTS** | | |  |
| Study selection | 16a | Describe the results of the search and selection process, from the number of records identified in the search to the number of studies included in the review, ideally using a flow diagram. | Pg 9-10 |
|  | 16b | Cite studies that might appear to meet the inclusion criteria, but which were excluded, and explain why they were excluded. | - |
| Study characteristics | 17 | Cite each included study and present its characteristics. | Pg 11-12, Table 1, Table 2 |
| Risk of bias in studies | 18 | Present assessments of risk of bias for each included study. | Figure 5 |
| Results of individual studies | 19 | For all outcomes, present, for each study: (a) summary statistics for each group (where appropriate) and (b) an effect estimate and its precision (e.g. confidence/credible interval), ideally using structured tables or plots. | Figure 5 |
| Results of syntheses | 20a | For each synthesis, briefly summarise the characteristics and risk of bias among contributing studies. | Pg 40 |
|  | 20b | Present results of all statistical syntheses conducted. If meta-analysis was done, present for each the summary estimate and its precision (e.g. confidence/credible interval) and measures of statistical heterogeneity. If comparing groups, describe the direction of the effect. | Pg 40, Figure 5 |
|  | 20c | Present results of all investigations of possible causes of heterogeneity among study results. | Figure 5 |
|  | 20d | Present results of all sensitivity analyses conducted to assess the robustness of the synthesized results. | Figure 5, Appendix IV |
| Reporting biases | 21 | Present assessments of risk of bias due to missing results (arising from reporting biases) for each synthesis assessed. | Pg 40, Figure 5, Appendix IV |
| Certainty of evidence | 22 | Present assessments of certainty (or confidence) in the body of evidence for each outcome assessed. | Appendix III |
| **DISCUSSION** | | |  |
| Discussion | 23a | Provide a general interpretation of the results in the context of other evidence. | Pg 43-46 |
|  | 23b | Discuss any limitations of the evidence included in the review. | Pg 46 |
|  | 23c | Discuss any limitations of the review processes used. | Pg 46 |
|  | 23d | Discuss implications of the results for practice, policy, and future research. | Pg 46-47 |
| **OTHER INFORMATION** | | |  |
| Registration and protocol | 24a | Provide registration information for the review, including register name and registration number, or state that the review was not registered. | Pg 48 |
|  | 24b | Indicate where the review protocol can be accessed, or state that a protocol was not prepared. | Pg 48 |
|  | 24c | Describe and explain any amendments to information provided at registration or in the protocol. | N/A |
| Support | 25 | Describe sources of financial or non-financial support for the review, and the role of the funders or sponsors in the review. | Pg 48 |
| Competing interests | 26 | Declare any competing interests of review authors. | Pg 48 |
| Availability of data, code and other materials | 27 | Report which of the following are publicly available and where they can be found: template data collection forms; data extracted from included studies; data used for all analyses; analytic code; any other materials used in the review. | Pg 48 |

*From:*  Page MJ, McKenzie JE, Bossuyt PM, Boutron I, Hoffmann TC, Mulrow CD, et al. The PRISMA 2020 statement: an updated guideline for reporting systematic reviews. BMJ 2021;372:n71. doi: 10.1136/bmj.n71

**Appendix III. Search strategy**

| **Search terms** | | | | |
| --- | --- | --- | --- | --- |
| **OVID- MEDLINE, PsycINFO and EMBASE** | | | | |
| Rural Health Services.mp. [mp=ti, ab, hw, tn, ot, dm, mf, dv, kf, fx, dq, nm, ox, px, rx, an, ui, sy, tc, id, tm, mh]  OR  Medically underserved area.mp. [mp=ti, ab, hw, tn, ot, dm, mf, dv, kf, fx, dq, nm, ox, px, rx, an, ui, sy, tc, id, tm, mh]  OR  (rural or remote or nonmetropolitan).mp. [mp=ti, ab, hw, tn, ot, dm, mf, dv, kf, fx, dq, nm, ox, px, rx, an, ui, sy, tc, id, tm, mh]  OR  ((rural or remote or nonmetropolitan or underserved or under served or deprived) adj (communit* or area* or region* or province*)).mp. [mp=ti, ab, hw, tn, ot, dm, mf, dv, kf, fx, dq, nm, ox, px, rx, an, ui, sy, tc, id, tm, mh]  OR  regional.mp. [mp=ti, ab, hw, tn, ot, dm, mf, dv, kf, fx, dq, nm, ox, px, rx, an, ui, sy, tc, id, tm, mh]  OR   (inequitable distribut* or maldistribut*).mp. [mp=ti, ab, hw, tn, ot, dm, mf, dv, kf, fx, dq, nm, ox, px, rx, an, ui, sy, tc, id, tm, mh]  OR  (rural adj (setting* or clinic* or communit* or hospital*)).mp. [mp=ti, ab, hw, tn, ot, dm, mf, dv, kf, fx, dq, nm, ox, px, rx, an, ui, sy, tc, id, tm, mh]  OR  rural health.mp. [mp=ti, ab, hw, tn, ot, dm, mf, dv, kf, fx, dq, nm, ox, px, rx, an, ui, sy, tc, id, tm, mh]  OR   rural population.mp. [mp=ti, ab, hw, tn, ot, dm, mf, dv, kf, fx, dq, nm, ox, px, rx, an, ui, sy, tc, id, tm, mh]  OR  Rural Health/  OR  Rural population/  OR  (Indigenous peoples or Indigenous or native or Aborig*).mp. [mp=ti, ab, hw, tn, ot, dm, mf, dv, kf, fx, dq, nm, ox, px, rx, an, ui, sy, tc, id, tm, mh]  OR  Indigenous peoples/  AND  program*.mp. [mp=ti, ab, hw, tn, ot, dm, mf, dv, kf, fx, dq, nm, ox, px, rx, an, ui, sy, tc, id, tm, mh]  OR  intervention*.mp. [mp=ti, ab, hw, tn, ot, dm, mf, dv, kf, fx, dq, nm, ox, px, rx, an, ui, sy, tc, id, tm, mh]  OR  internet-based intervention/  OR  health education/  OR   education*.mp. [mp=ti, ab, hw, tn, ot, dm, mf, dv, kf, fx, dq, nm, ox, px, rx, an, ui, sy, tc, id, tm, mh]  OR  behav* intervention.mp. [mp=ti, ab, hw, tn, ot, dm, mf, dv, kf, fx, dq, nm, ox, px, rx, an, ui, sy, tc, id, tm, mh]  OR   health promotion*.mp. [mp=ti, ab, hw, tn, ot, dm, mf, dv, kf, fx, dq, nm, ox, px, rx, an, ui, sy, tc, id, tm, mh]  OR  patient education.mp. [mp=ti, ab, hw, tn, ot, dm, mf, dv, kf, fx, dq, nm, ox, px, rx, an, ui, sy, tc, id, tm, mh]  OR  OR  Treatment/  Or  Therapeutics/  Or  treat*.mp. [mp=ti, ab, hw, tn, ot, dm, mf, dv, kf, fx, dq, nm, ox, px, rx, an, ui, sy, tc, id, tm, mh]  OR therapeutics/  OR  Public health  OR  Initiative* | | | | |
| **Risk factors** | Physical activity | Depression | Social Isolation | Diet |
|  | AND  (physical activity or exercise* or physical inactivity or sedentary behav*).mp. [mp=ti, ab, hw, tn, ot, dm, mf, dv, kf, fx, dq, nm, ox, px, rx, an, ui, sy, tc, id, tm, mh]  OR  Exercise/  OR  Exercise movement techniques/  OR   exercise therapy/  OR  Sedentary behaviour/  OR  (sport* or athletic participation or exercise behav* or walk* or fitness).mp. [mp=ti, ab, hw, tn, ot, dm, mf, dv, kf, fx, dq, nm, ox, px, rx, an, ui, sy, tc, id, tm] | AND  (depression or anxiety).mp. [mp=ti, ab, hw, tn, ot, dm, mf, dv, kf, fx, dq, nm, ox, px, rx, an, ui, sy, tc, id, tm, mh]  OR   anxiety disorders/  OR  mood disorders/  OR  depressive disorder.mp. [mp=ti, ab, hw, tn, ot, dm, mf, dv, kf, fx, dq, nm, ox, px, rx, an, ui, sy, tc, id, tm, mh]  OR  depressive disorder, Major/ | AND  Social isolation/  OR  Social environment/  OR  (social isolation or social alienation or loneliness or social connect* or isolation or social participation).mp. [mp=ti, ab, hw, tn, ot, dm, mf, dv, kf, fx, dq, nm, ox, px, rx, an, ui, sy, tc, id, tm, mh]  OR  (Social*). mp. [mp=ti, ab, hw, tn, ot, dm, mf, dv, kf, fx, dq, nm, ox, px, rx, an, ui, sy, tc, id, tm, mh] | AND  Diet/  exp Diet/  Diet, Mediterranean/  Dietary pattern.mp. [mp=ti, ab, hw, tn, ot, dm, mf, dv, kf, fx, dq, bt, nm, ox, px, rx, an, ui, sy, ux, mx]  Medi diet.mp. [mp=ti, ab, hw, tn, ot, dm, mf, dv, kf, fx, dq, bt, nm, ox, px, rx, an, ui, sy, ux, mx]  MedDiet.mp. [mp=ti, ab, hw, tn, ot, dm, mf, dv, kf, fx, dq, bt, nm, ox, px, rx, an, ui, sy, ux, mx]  Mediterranean dietary pattern.mp. [mp=ti, ab, hw, tn, ot, dm, mf, dv, kf, fx, dq, bt, nm, ox, px, rx, an, ui, sy, ux, mx]  diet.mp. [mp=ti, ab, hw, tn, ot, dm, mf, dv, kf, fx, dq, bt, nm, ox, px, rx, an, ui, sy, ux, mx]  dietary intake.mp. [mp=ti, ab, hw, tn, ot, dm, mf, dv, kf, fx, dq, bt, nm, ox, px, rx, an, ui, sy, ux, mx]  dietary quality.mp. [mp=ti, ab, hw, tn, ot, dm, mf, dv, kf, fx, dq, bt, nm, ox, px, rx, an, ui, sy, ux, mx]  dietary behav*.mp. [mp=ti, ab, hw, tn, ot, dm, mf, dv, kf, fx, dq, bt, nm, ox, px, rx, an, ui, sy, ux, mx]  nutrition.mp. [mp=ti, ab, hw, tn, ot, dm, mf, dv, kf, fx, dq, bt, nm, ox, px, rx, an, ui, sy, ux, mx]  food insecurity.mp. [mp=ti, ab, hw, tn, ot, dm, mf, dv, kf, fx, dq, bt, nm, ox, px, rx, an, ui, sy, ux, mx]  dietary advice.mp. [mp=ti, ab, hw, tn, ot, dm, mf, dv, kf, fx, dq, bt, nm, ox, px, rx, an, ui, sy, ux, mx]  cretan diet.mp. [mp=ti, ab, hw, tn, ot, dm, mf, dv, kf, fx, dq, bt, nm, ox, px, rx, an, ui, sy, ux, mx]  exp fruit/  exp vegetables/  exp seafood/  red meat.mp. [mp=ti, ab, hw, tn, ot, dm, mf, dv, kf, fx, dq, bt, nm, ox, px, rx, an, ui, sy, ux, mx]  exp fatty acids/  exp omega-3/  exp dietary fats/  Nutrient*.mp. AND Consum*.mp. [mp=ti, ab, hw, tn, ot, dm, mf, dv, kf, fx, dq, bt, nm, ox, px, rx, an, ui, sy, ux, mx]  intake.mp OR ingest*.mp.[mp=ti, ab, hw, tn, ot, dm, mf, dv, kf, fx, dq, bt, nm, ox, px, rx, an, ui, sy, ux, mx]  OR  cooking .mp. [mp=ti, ab, hw, tn, ot, dm, mf, dv, kf, fx, dq, bt, nm, ox, px, rx, an, ui, sy, ux, mx]  OR  exp fatty acids/ OR Essential/  OR  Exp fatty acids/ OR Monounsaturated  OR  Unsaturated/ OR trans fatty acids/  Or consum* OR intake or ingest* [mp=ti, ab, hw, tn, ot, dm, mf, dv, kf, fx, dq, bt, nm, ox, px, rx, an, ui, sy, ux, mx] |
| Limit to "middle age (45 to 64 years)" or "middle aged (45 plus years)" or "all aged (65 and over)" or "aged (80 and over)" or 360 middle age <age 40 to 64 yrs> or "380 aged <age 65 yrs and older>" or "390 very old <age 85 yrs and older>"  Limit to yr= “2000-current”  Limit to humans  Limit to full-text  English | | | | |
| **Cochrane Central Register of Controlled Trials** | | | | |
| **Trials only** | **Search Name: Physical activity Interventions in rural older adults**  ID Search Hits  #1 MeSH descriptor: [Rural Population] this term only 1850  #2 MeSH descriptor: [Rural Health] this term only 535  #3 ((rural OR regional OR remote) NEAR/3 (health or population or communit*)):ti,ab,kw 5235  #4 ((rural or remote or nonmetropolitan or underserved or under served or deprived) adj (communit* or area* or region* or province*)) 555  #5 (rural adj (setting* or clinic* or hospital*)) 265  #6 MeSH descriptor: [Rural Health Services] explode all trees 355  #7 (rural or remote or nonmetropolitan or region* or province):ti,ab,kw 70638  #8 MeSH descriptor: [Medically Underserved Area] explode all trees 131  #9 #1 OR #2 OR #3 OR #4 OR #5 OR #6 OR #7 OR #8 71195  #10 MeSH descriptor: [Aged] explode all trees 215798  #11 MeSH descriptor: [Middle Aged] this term only 327574  #12 ("elderly"):ti,ab,kw 52446  #13 ("older adult"):ti,ab,kw 991  #14 ("senior" NEAR/2 (older or adult or population)):ti,ab,kw 62  #15 ("geriatric"):ti,ab,kw 8130  #16 (program*):ti,ab,kw 135345  #17 (intervention*):ti,ab,kw 452927  #18 (behav* intervention):ti,ab,kw 63839  #19 MeSH descriptor: [Internet-Based Intervention] this term only 266  #20 MeSH descriptor: [Health Education] explode all trees 20884  #21 (treat*):ti,ab,kw 909790  #22 (Education*):ti,ab,kw 87235  #23 MeSH descriptor: [Patient Education as Topic] this term only 9132  #24 MeSH descriptor: [Therapeutics] this term only 69  #25 MeSH descriptor: [Public Health] explode all trees 456472  #26 MeSH descriptor: [Exercise Therapy] 1 tree(s) exploded 15285  #27 MeSH descriptor: [Exercise Movement Techniques] explode all trees 2348  #28 (physical activity):ti,ab,kw 45788  #29 (physical inactivity):ti,ab,kw 1781  #30 (sport or athletic participation or exercise behav* or walk*):ti,ab,kw 49792  #31 #26 OR #27 #28 OR #29 OR #30 61644  #32 #10 OR #11 #12 OR #13 OR #14 OR #15 221578  #33 #16 OR #17 OR #18 OR #19 OR #20 OR #21 OR #22 OR #23 OR #24 or #25 1311919  #34 #9 AND #31 AND #32 AND #33 with Publication Year from 2000 to 2021, with Cochrane Library publication date Between Jan 2000 and Dec 2021, in Trials | **Search Name: Depression Interventions in rural older adults**  ID Search Hits  #1 MeSH descriptor: [Rural Population] this term only 1850  #2 MeSH descriptor: [Rural Health] this term only 535  #3 ((rural OR regional OR remote) NEAR/3 (health or population or communit*)):ti,ab,kw 5235  #4 ((rural or remote or nonmetropolitan or underserved or under served or deprived) adj (communit* or area* or region* or province*)) 555  #5 (rural adj (setting* or clinic* or hospital*)) 265  #6 MeSH descriptor: [Rural Health Services] explode all trees 355  #7 (rural or remote or nonmetropolitan or region* or province):ti,ab,kw 70638  #8 MeSH descriptor: [Medically Underserved Area] explode all trees 131  #9 #1 OR #2 OR #3 OR #4 OR #5 OR #6 OR #7 OR #8 71195  #10 MeSH descriptor: [Aged] explode all trees 215798  #11 MeSH descriptor: [Middle Aged] this term only 327574  #12 ("elderly"):ti,ab,kw 52446  #13 ("older adult"):ti,ab,kw 991  #14 ("senior" NEAR/2 (older or adult or population)):ti,ab,kw 62  #15 ("geriatric"):ti,ab,kw 8130  #16 (program*):ti,ab,kw 135345  #17 (intervention*):ti,ab,kw 452927  #18 (behav* intervention):ti,ab,kw 63839  #19 MeSH descriptor: [Internet-Based Intervention] this term only 266  #20 MeSH descriptor: [Health Education] explode all trees 20884  #21 (treat*):ti,ab,kw 909790  #22 (Education*):ti,ab,kw 87235  #23 MeSH descriptor: [Patient Education as Topic] this term only 9132  #24 MeSH descriptor: [Therapeutics] this term only 69  #25 MeSH descriptor: [Public Health] explode all trees 456472  #26 MeSH descriptor: [Depression] this term only 13312  #27 MeSH descriptor: [Anxiety] this term only 8078  #28 MeSH descriptor: [Mood Disorders] explode all trees 13595  #29 MeSH descriptor: [Depressive Disorder, Major] this term only 5330  #30 MeSH descriptor: [Anxiety Disorders] this term only 4143  #31 (depression or anxiety):ti,ab,kw 111353  #32 #10 OR #11 #12 OR #13 OR #14 OR #15 221578  #33 #16 OR #17 OR #18 OR #19 OR #20 OR #21 OR #22 OR #23 OR #24 OR #25 1311919  #34 #26 or #27 or #28 or #29 Or #30 or #31 112905  #35 #9 AND #32 AND #33 AND #34 with Publication Year from 2000 to 2021, with Cochrane Library publication date Between Jan 2000 and Dec 2021, in Trials | **Search Name: Social isolation Interventions in rural older adults**  ID Search Hits  #1 MeSH descriptor: [Rural Population] this term only 2576  #2 MeSH descriptor: [Rural Health] this term only 610  #3 ((rural OR regional OR remote) NEAR/3 (health or population or communit*)):ti,ab,kw 6301  #4 ((rural or remote or nonmetropolitan or underserved or under served or deprived) adj (communit* or area* or region* or province*)) 611  #5 (rural adj (setting* or clinic* or hospital*)) 289  #6 MeSH descriptor: [Rural Health Services] explode all trees 416  #7 (rural or remote or nonmetropolitan or region* or province):ti,ab,kw 84552  #8 MeSH descriptor: [Medically Underserved Area] explode all trees 159  #9 #1 OR #2 OR #3 OR #4 OR #5 OR #6 OR #7 OR #8 85167  #10 MeSH descriptor: [Aged] explode all trees 256032  #11 MeSH descriptor: [Middle Aged] this term only 369181  #12 ("elderly"):ti,ab,kw 59003  #13 ("older adult"):ti,ab,kw 1306  #14 ("senior" NEAR/2 (older or adult or population)):ti,ab,kw 63  #15 ("geriatric"):ti,ab,kw 9502  #16 (program*):ti,ab,kw 160014  #17 (intervention*):ti,ab,kw 572336  #18 (behav* intervention):ti,ab,kw 78269  #19 MeSH descriptor: [Internet-Based Intervention] this term only 571  #20 MeSH descriptor: [Health Education] explode all trees 24480  #21 (treat*):ti,ab,kw 1029076  #22 (Education*):ti,ab,kw 105015  #23 MeSH descriptor: [Patient Education as Topic] this term only 10019  #24 MeSH descriptor: [Therapeutics] this term only 113  #25 MeSH descriptor: [Public Health] explode all trees 605603  #26 (initiative*):ti,ab,kw 6705  #27 #10 OR #11 #12 OR #13 OR #14 OR #15 262246  #28 #16 OR #17 OR #18 OR #19 OR #20 OR #21 OR #22 OR #23 OR #24 OR #25 OR #26 1545474  #29 #9 AND #27 AND #28 with Publication Year from 2000 to 2021, with Cochrane Library publication date Between Jan 2000 and Dec 2021, in Trials 8201  #30 MeSH descriptor: [Social Isolation] explode all trees with Publication Year from 2000 to 2021, with Cochrane Library publication date Between Jan 2000 and Dec 2021, in Trials 450  #31 MeSH descriptor: [Loneliness] this term only 254  #32 (Social connect*):ti,ab,kw 2594  #33 (social engage*):ti,ab,kw 5747  #34 (social network):ti,ab,kw 2924  #35 (isolation):ti,ab,kw 15972  #36 (social participation):ti,ab,kw 6395  #37 #30 OR #31 OR #32 OR #35 OR #33 OR #34 OR #35 OR #36 30388  #38 #29 and #37  Publication Year from 2000 to 2021, with Cochrane Library publication date Between Jan 2000 and Dec 2021, in Trials | **Search Name: Diet Interventions in rural older adults**  ID Search Hits  #1 MeSH descriptor: [Rural Population] this term only 2576  #2 MeSH descriptor: [Rural Health] this term only 610  #3 ((rural OR regional OR remote) NEAR/3 (health or population or communit*)):ti,ab,kw 6301  #4 ((rural or remote or nonmetropolitan or underserved or under served or deprived) adj (communit* or area* or region* or province*)) 611  #5 (rural adj (setting* or clinic* or hospital*)) 289  #6 MeSH descriptor: [Rural Health Services] explode all trees 416  #7 (rural or remote or nonmetropolitan or region* or province):ti,ab,kw 84551  #8 MeSH descriptor: [Medically Underserved Area] explode all trees 159  #9 #1 OR #2 OR #3 OR #4 OR #5 OR #6 OR #7 OR #8 85166  #10 MeSH descriptor: [Aged] explode all trees 256032  #11 MeSH descriptor: [Middle Aged] this term only 369181  #12 ("elderly"):ti,ab,kw 59003  #13 ("older adult"):ti,ab,kw 1306  #14 ("senior" NEAR/2 (older or adult or population)):ti,ab,kw 63  #15 ("geriatric"):ti,ab,kw 9502  #16 (program*):ti,ab,kw 160016  #17 (intervention*):ti,ab,kw 572336  #18 (behav* intervention):ti,ab,kw 78269  #19 MeSH descriptor: [Internet-Based Intervention] this term only 571  #20 MeSH descriptor: [Health Education] explode all trees 24480  #21 (treat*):ti,ab,kw 1029077  #22 (Education*):ti,ab,kw 105015  #23 MeSH descriptor: [Patient Education as Topic] this term only 10019  #24 MeSH descriptor: [Therapeutics] this term only 113  #25 MeSH descriptor: [Public Health] explode all trees 605603  #26 (initiative*):ti,ab,kw 6705  #27 #10 OR #11 #12 OR #13 OR #14 OR #15 262246  #28 #16 OR #17 OR #18 OR #19 OR #20 OR #21 OR #22 OR #23 OR #24 OR #25 OR #26 1545475  #29 #9 AND #27 AND #28 with Publication Year from 2000 to 2023, with Cochrane Library publication date Between Jan 2000 and Nov 2023, in Trials 8789  #30 MeSH descriptor: [Diet, Food, and Nutrition] explode all trees 73842  #31 MeSH descriptor: [Diet, Mediterranean] this term only 786  #32 (Dietary pattern):ti,ab,kw 2389  #33 (Medi diet):ti,ab,kw 297  #34 (MedDiet):ti,ab,kw 249  #35 (Mediterranean diet):ti,ab,kw 2492  #36 (dietary intake):ti,ab,kw 26931  #37 (dietary behav*):ti,ab,kw 9152  #38 ("nutrition education research"):ti,ab,kw 2  #39 ("nutrition support service"):ti,ab,kw 5  #40 (food insecurity):ti,ab,kw 642  #41 (dietary advice):ti,ab,kw 2495  #42 (cretan diet):ti,ab,kw 14  #43 MeSH descriptor: [Fruit] explode all trees 3541  #44 MeSH descriptor: [Vegetables] explode all trees 2265  #45 MeSH descriptor: [Seafood] explode all trees 251  #46 (red meat):ti,ab,kw 683  #47 MeSH descriptor: [Fatty Acids] explode all trees 26108  #48 MeSH descriptor: [Fatty Acids, Omega-3] explode all trees 3831  #49 MeSH descriptor: [Fatty Acids, Omega-6] explode all trees 867  #50 MeSH descriptor: [Dietary Fats] explode all trees 8815  #51 (Nutrient):ti,ab,kw 6710  #52 (Nutrient AND Consum*):ti,ab,kw 2095  #53 (Intake OR ingest):ti,ab,kw 67040  #54 (cooking):ti,ab,kw 1719  #55 MeSH descriptor: [Fatty Acids, Essential] explode all trees 2638  #56 MeSH descriptor: [Fatty Acids, Monounsaturated] explode all trees 3075  #57 MeSH descriptor: [Fatty Acids, Unsaturated] explode all trees 14437  #58 MeSH descriptor: [Trans Fatty Acids] explode all trees 60  #59 (consum* intake or ingest*):ti,ab,kw 36749  #60 #30 OR #31 OR #32 OR #33 OR #34 OR #35 OR ##6 OR #37 OR #38 OR #39 OR #40 OR #41 OR #42 OR #43 OR #44 OR #45 OR # 46 OR #47 OR #48 OR #49 OR #50 OR #51 OR #52 OR #53 OR #54 OR #55 OR #56 OR #57 OR #58 OR #59 252038  #61 #60 AND #29 with Publication Year from 2000 to 2023, with Cochrane Library publication date Between Jan 2000 and Nov 2023, in Trials |
| **Web of Science: core collection** | | | | |
| (((((TS=(aged)) OR TS=(elderly*)) OR TS=(older adult*)) OR TS=(aged 80 and over)) OR TS=(older senior*)) OR TS=(geriatric) OR TS=(middle-age*) OR TS=(middle age*)  AND  TS=(Rural Health Service* OR Medically underserved area OR rural OR remote OR regional OR underserved OR under served OR (inequitable distribut* or maldistribut*) OR (rural adj (setting* or clinic* or communit* or hospital*)) OR rural health OR rural population* OR Indigenous peoples OR Indigenous OR native OR Aborig*)  OR  TS=((rural or remote or nonmetropolitan or underserved or under served or deprived) adj (communit* or area* or region* or province*))  AND TS=(program* or intervention* or internet-based intervention or health education or education* or behav* intervention or health promotion* or patient education or treatment or treat* or therapeutics or public health or initiative*) | | | | |
| **Risk factors** | Physical activity | Depression | Social Isolation | Diet  Conducted on 10/11/23  Filter by date range:  01-01-2000 to 10-11-2023  Refine to articles and English only |
|  | AND  TS=(Physical activit* or exercise* or physical inactivit* or sedentary behav* or sport or athletic participation or exercise behav* or walk*) | AND  TS=(depression or anxiety or anxiety disorder* or mood disorder* or depressive disorder* or major depressive disorder) | TS=(social isolation or loneliness or lonely or social environment* or social alienation or social connect* or isolation or social participation*) | TS=(Diet OR Diet, Mediterranean OR Dietary pattern OR Mediterranean dietary pattern OR dietary intake OR dietary quality OR dietary behav* OR nutrition OR food insecurity OR dietary advice) |
| Limit to yr= “2000-current”  English  Journal articles | | | | |
| **CINAHL** | | | | |
| MH ( adults or adult or middle aged ) OR MH ( aged, 80 and over ) OR MH ( aged or elderly or senior or older people or geriatric ) OR MH ( older adults or elderly or aged 65 or 65+ )  AND  MH ( rural areas or rural communities ) OR MH ( remote area or isolated community or rural region ) OR MH ( nonmetropolitan or region ) OR MH underserved populations OR MH province OR MH ( rural settings or rural population or rural health )  OR  MH indigenous peoples OR MH indigenous OR MH ( indigenous or native or aboriginal or indians or first nations ) OR MH ( indigenous australians or aboriginal australians or torres straight islanders ) OR MH indigenous health  AND  MH program OR MH ( interventions or strategies or best practices or treatment or therapy or program or management ) OR MH behaviour change OR MH ( health education or health promotion or health teaching or patient education or health literacy ) OR MH public health OR MH initiative | | | | |
| **Risk factors** | Physical activity | Depression | Social isolation | Diet  9/11/23 |
|  | AND  ( MH (exercise or physical activity or fitness or physical exercise) OR MH ( exercise or physical activity or fitness or aerobic training or strength training or cardiovascular training ) OR MH ( exercise therapy or physical therapy or exercise intervention ) OR MH ( physical inactivity or physically inactive or sedentary or lack of physical activity ) OR MH exercise movement techniques ) OR MH athletic participation OR MH ( walking or exercise or physical activity ) OR MH ( walking program or walking intervention or walking ) OR MH sports | AND MH ( depression and anxiety ) OR MH ( depression or depressive disorder or depressive symptoms or major depressive disorder ) OR MH mood disorders OR anxiety disorders | AND MH ( social isolation or loneliness or social exclusion or social deprivation ) OR MH ( social environment or social support or social relationships ) OR MH social alienation OR MH ( social connection or connectedness or community connectedness ) | MH diet/ OR MH exp diet/ OR MH diet, Mediterranean/ OR MH ( Dietary pattern OR MedDiet OR Mediterranean dietary pattern OR diet OR dietary quality OR dietary behav* OR nutrition OR food insecurity OR dietary advice ) OR MH exp fruit/ OR MH exp vegetables/ OR MH exp seafood/ OR MH red meat OR MH exp fatty acids/ OR MH exp omega-3/ OR MH exp dietary fats/ OR MH nutrient* OR  MH ( intake OR ingest* ) OR MH cooking OR MH ( exp fatty acids/ OR Essential/ ) OR MH ( Exp fatty acids/ OR Monounsaturated ) OR MH ( Unsaturated/ OR trans fatty acids/ ) OR TH ( consump* OR intake OR ingest* ) |
| Limit to: Full Text; Age Groups: Middle Aged: 45-64 years, Aged: 65+ years, Aged, 80 and over; Published Date: 20000101-20211231; English Language; Peer Reviewed | | | | |

**Appendix IV. GRADE assessment**

| **GRADE** | | | | | | | | | **No. of participants** |  | **Effect** | **Effect size** | **Certainty** |
| --- | --- | --- | --- | --- | --- | --- | --- | --- | --- | --- | --- | --- | --- |
| **Risk factors** | **Outcomes** | **Study Design** | **No.** | **Risk of bias** | **Inconsistency** | **Indirectness** | **Imprecision** | **Publication bias** | **Intervention** | **Control** |  |  |  |
| Depression | Depressive symptoms | Randomised Controlled trials | 7 | Serious limitations ^*^ | Not serious | Serious limitations^\|\|^ | Not serious | Not serious (undetected) | 1545 | 1438 | -0.39 [-0.55,  -0.24] | **Moderate** | Moderate |
| Social isolation | Loneliness | Randomised Controlled trials | 2 | No serious limitations | Serious limitations^‡^ | Serious limitations^\|\|^ | Serious limitations^{^ | Not serious (undetected) | 94 | 48 | -4.83 [-12.55, 2.89] | - | Low |
| Physical inactivity | Physical function | Randomised Controlled trials | 6 | Serious limitations ^*,†^ | Serious limitations§ | Very serious limitations^\|\|^ | Serious limitations^#^ | Not serious (undetected) | 176 | 163 | -0.03 [-1.15, 1.10] | **Small** | Very low |

| * Inadequate randomisation, and blinding of participants, personnel and outcome assessment |
| --- |
| † Unclear selective reporting |
| ‡ Considerable heterogeneity is observed (75-100%), p value is low, CI do not overlap |
| § Considerable heterogeneity is observed (75-100%), p value is low |
| \|\| Indirectness as a result of differences in comparison/control groups and differences in intervention length/follow-up time points. |
| { Imprecision due to small sample size and confidence intervals large |
| # Indirectness as a result of differences in comparison/control groups and differences in intervention length/follow-up time points, and use of surrogate outcomes (e.g. Step count for physical activity) |

**Appendix V. Funnel plot with data from a) physical inactivity interventions, b) social isolation interventions, and c) depression interventions**

| **a)** | **b)** |
| --- | --- |
| 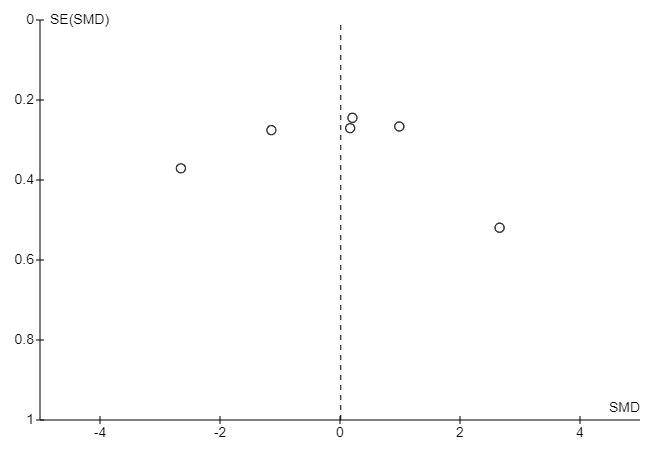 | 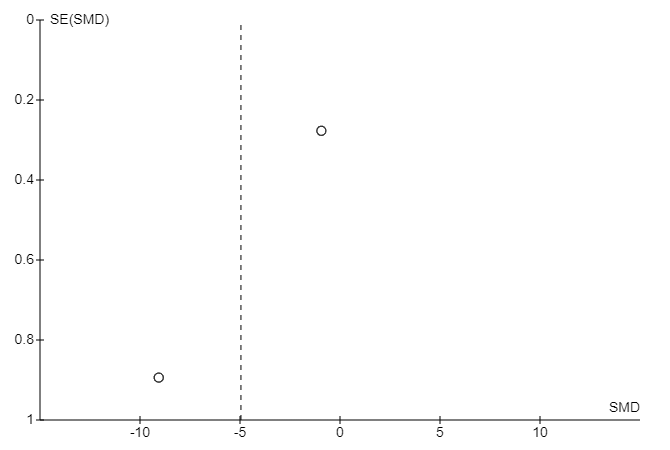 |
| **c)** |  |
| 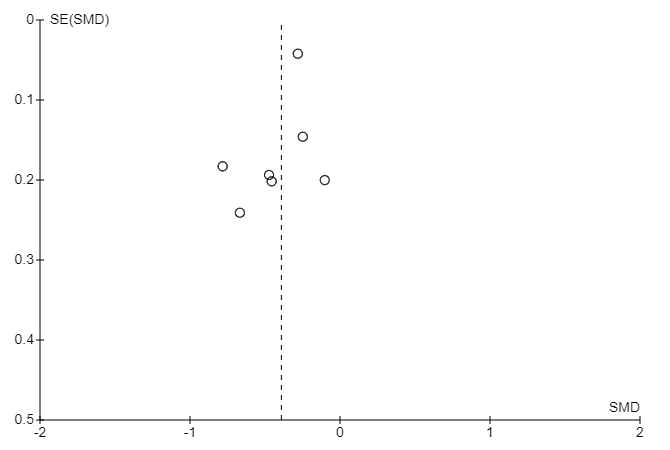 | |

**Appendix VI. Detailed summary of primary and secondary intervention outcomes for each risk factor**

**Physical inactivity (N=22)**

| **Author (year)** | **Data Collection Time Points** | **Outcome** | | | | | | |
| --- | --- | --- | --- | --- | --- | --- | --- | --- |
|  |  | **Physical function (O) ^*^** | **Physical and mental wellbeing (S) ^†^** | **Physical activity (O) ^‡^** | **Physical activity (S) ^§^** | **Anthropometrics (O) ^\|\|^** | **Self-efficacy (S)^{^** | **Other^#^** |
| Akihiro (2018) [76] | 2 (Baseline, 3 months (end of intervention)) | X^short^ | X^short^ |  |  |  |  |  |
| Britten (2023) [82] | 4 (Baseline, 3 months, 6 months, 12 months) |  | X^short, long^ |  | X^short, long^ |  |  |  |
| Cai (2022) [77] | 2 (Baseline, 3 months) |  |  | X^short^ |  |  | X^short^ |  |
| Cruz-Ferreira (2015) [78] | 3 (Baseline, 3 months, 6 months) |  | X^short^ | X^short^ |  |  |  |  |
| Harris (2015) [71] | 3 (Baseline, 3 months, 12 months) |  | X | X^short, long^ |  | X |  |  |
| Jang (2018) [83] | 2 (0, 6 months, 13 months) | X^short^ | X^short^ |  | X^short^ | X^short^ |  | X^short^ |
| Jeon (2014) [79] | 2 (0, 12 weeks) | X^short^ | X^short^ |  |  |  | X^short^ | X^short^ |
| Jindo (2017) [84] | 2 (0, 11 weeks) | X^short^ |  | X |  |  |  |  |
| Johnson (2017) [80] | 2 (0, 6 months) | X^short^ | X |  |  |  |  |  |
| Jones (2023) [85] | 3 (Baseline, 16 weeks, 32 weeks) | X^short, long^ | X^short^ |  |  |  | X^short, long^ | X^short^ |
| Joo (2019) [86] | 3 (0, 6 months, 12 months) | X^long^ |  |  |  | X^long^ |  |  |
| Kim (2021) [87] | 2 (0, 7 months) | X^long^ | X^long^ |  |  | X^long^ |  |  |
| Kleinke (2021) [68] | 3 (0, 3 months, 6 months) |  |  | X |  |  |  |  |
| Lee (2007) [69] | 2 (0, 6 months) |  |  |  | X^short^ | X^short^ | X^short^ |  |
| Mcmahon (2016) [81] | 2 (0, 1 week) |  |  | X^short^ | X^short^ |  |  | X^short^ |
| Mcnamara (2023) [88] | 2 (Baseline, 9 weeks) | X^short^ | X^short^ |  |  |  |  |  |
| O’Brien (2016) [70] | 4 (0, 4 weeks, 8 weeks, 12 weeks) |  |  |  | X | X | X | X |
| Park (2015) [89] | 2 (0, 8 weeks) | X^short^ | X^short^ |  |  |  |  | X |
| Paschoa (2016) [90] | 2 (0, 9 weeks) |  |  |  | X^short^ | X^short^ |  | X^short^ |
| Sowle (2017) [91] | 2 (0, 8 weeks) |  |  |  | X^short^ |  | X |  |
| Strand (2014) [92] | 3 (0, 8 weeks, 25 weeks) |  | X |  | X^short^ |  |  | X |
| Yeh (2022) [93] | 2 (0, 3 months) | X^short^ | X^short^ |  |  | X^short^ |  |  |

X indicates at least one measure of that outcome is significant

^short^ Significant short-term outcome/s

^long^ Significant long-term outcome/s

(S) Self report measures were used

(O) Objective measures were used

^*^Physical function (O) includes mobility, balance, grip strength, gait speed

^†^Physical and mental wellbeing includes PROMIS general health questionnaire, life satisfaction, health state, depression, anxiety, pain, quality of life, psychologic status

^‡^Physical activity (O) includes physical fitness
^§^Physical activity (S) includes walking frequency, knowledge of and importance of an active lifestyle, self reported physical activity questionnaire, goal attainment, readiness to change

^||^Anthropometrics includes body composition measures (weight, height, BMI, fat mass, BP, waist to hip ratio heart rate), cardiovascular health related factors (HbA1c, HDLc, LDLc, TC, Triglycerides), lung function (forced vital capacity).

^{^ Self-efficacy includes physical activity self-efficacy, falls self-efficacy, exercise self-efficacy, eating behaviour self-efficacy

^#^ Other includes process outcomes include feasibility and acceptability, wearable device adherence, falls risk, theoretical mechanisms of change, falls preventative behaviours, fear of falling, knowledge of importance of active lifestyle, satisfaction with program

**Poor diet (N=6)**

| **Author (year)** | **Data Collection Time Points** | **Outcomes** | | | | | | | |
| --- | --- | --- | --- | --- | --- | --- | --- | --- | --- |
|  |  | **Diet Quality (S)^*^** | **Diet Behaviour (S)^†^** | **Anthropometrics (O) ^‡^** | **SBP^§^** | **DBP^\|\|^** | **Perceived hypertension severity (S)** | **Perceived self-efficacy on dietary behaviours (S)** | **Other^**^** |
| Al-Nimr (2020) [72] | 2 (Baseline, 12 weeks) | X^short^ |  | X^short^ |  |  |  |  |  |
| Goni (2020) [73] | 3 (Baseline, 12 months, 24 months) | X^long^ |  |  |  |  |  |  |  |
| Hamirudin (2017) [94] | 2 (Baseline, 3 months) | X^short^ |  | X^short^ |  |  |  |  |  |
| Sachdeva (2008) [95] | 2 (Baseline, 12 weeks) |  | X^short^ |  |  |  |  |  |  |
| Seangpraw (2019) [74] | 3(Baseline, 3 months, 6 months) |  |  |  | X^short^ | X^short^ | X^short^ | X^short^ |  |
| Shahar (2012) [96] | 2 (Baseline, 3months, 6 months) |  |  | X^short^ |  | X^short^ |  |  |  |

X indicates at least one measure of that outcome is significant

^short^Signficant short-term outcome/s

^long^Signficant long-term outcome/s

(S) Self report measures were used

(O) Objective measure were used

^*^Diet quality includes Rapid Eating Activity Assessment for Patients-short version, Automated self-administered 24 hr dietary recall survey, Mediterranean diet screener, Food frequency Questionnaire

^†^Diet behaviour includes Knowledge, attitudes and practices survey

^‡^Anthropometrics includes weight and waist circumference, BMI, fasting blood glucose, HDLc, LDLc, TC, Triglycerides

^§^SBP is Systolic Blood Pressure

^||^DBP is Diastolic Blood pressure

^{^Physical function includes gait speed, grip strength

^#^Physical and mental wellbeing includes PROMIS General Health Questionnaire
^**^Other includes process outcomes like feasibility and acceptability

**Social isolation (N=9)**

| **Study** | **Data Collection Time Points** | **Outcomes** | | | | | |
| --- | --- | --- | --- | --- | --- | --- | --- |
|  |  | **Social networks (S)^*^** | **Physical function (O)^†^** | **Loneliness (S)^‡^** | **Self-efficacy (S)^§^** | **Mental wellbeing (S) ^\|\|^** | **Other^{^** |
| Ashida (2017) [99] | 2 (0, 1 month) | X^short^ |  |  |  |  |  |
| Banbury (2017) [100] | 2 (0, 5 months) |  |  |  |  |  |  |
| Kikuchi (2023) [101] | 2 (0, 3 months) |  | X^short^ | X^short^ | X^short^ |  |  |
| Kim (2020) [102] | 2 (0, 10 weeks) | X^short^ |  | X^short^ | X | X^short^ |  |
| Kim (2021) [103] | 2 (0, 13 months) | X |  | X |  | X^long^ |  |
| Li (2019) [97] | 3 (0, 8 months, 11 months) |  |  | X^long^ |  | X^long^ |  |
| Oetzel (2020) [104] | 3 (0 months, 4 months, 8 months) |  |  | X | X^long^ | X^long^ | X^long^ |
| Shapira (2020) [98] | 2 (0, 3.5 weeks) |  |  | X^short^ |  | X^short^ |  |
| Willard (2018) [105] | 2 (0, 4 months) | X |  |  |  |  | X^short^ |

X indicates at least one measure of that outcome is significant

^short^Significant short-term outcome/s

^long^Significant long-term outcome/s

(S) Self report measures were used

(O) Objective measure were used

^*^Social networks includes social support, emergency support network, social participation attitude, satisfaction with the way of spending time in social activities, community involvement (use of online platform)

^†^Physical function includes balance

^‡^Loneliness includes UCLA Loneliness scale, Kodama Loneliness scale

^§^Self-efficacy (S) includes ego integration, role self-efficacy, self-efficacy, autonomy, self actualisation

^||^Mental and physical wellbeing (S) includes Health, depressive symptoms, self-esteem, perceived stress, self-rated health, Health-related Quality of Life, spirituality, perceived support, desired support, subjective health, subjective wellbeing, life satisfaction

^{^Other includes likelihood of seeking services, burden, benefit, tribal identity, knowledge of tikanga, missed bill payments, house problems; use of tech and involvement in community (information provision, giving and seeking help from the community), expectations of intervention, perceived impact (consulting care or welfare services)

**Depression (N=12)**

| **Author (year)** | **Data Collection Time Points** | **Outcomes** | | | | | | | |
| --- | --- | --- | --- | --- | --- | --- | --- | --- | --- |
|  |  | **Anxiety (S)^*^** | **Depression (S) ^†^** | **Hypertension (O) ^‡^** | **Wellbeing and quality of life (S) ^§^** | **Self-efficacy (S) ^\|\|^** | **Social network (S) ^{^** | **Cognition and function (S,O)^#^** | **Other^**^** |
| Brenes (2015) [106] | 3 (Baseline, 2 months, 4 months) | X^short^ | X^short^ |  |  |  |  |  |  |
| Chen (2022) [107] | 5 (Baseline, 3 months, 6 months, 9 months, 12 months) |  | X^short, long^ | X^long^ |  |  |  |  |  |
| Chojak (2023) [108] | 2 (Baseline, 12 weeks) | X^short^ | X^short^ |  | X^short^ |  |  |  |  |
| Haringsma (2006) [109] | 4 (Baseline, 10 weeks, 2 months, 14 months) | X^short, long^ | X^short, long^ |  | X |  |  |  |  |
| Hollister (2022) [113] | 6 (Baseline, 3,6,9,12, 24 weeks) |  | X^short^ |  |  | X |  |  | X^short, long^ |
| Kim (2021) [103] | 2 (Baseline, 10 weeks) |  | X^short^ |  | X^short^ | X | X^short^ |  |  |
| Oh (2020) [75] | 2 (Baseline, 12 weeks) |  | X^short^ |  |  |  | X^short^ | X | X^short^ |
| Sarkar (2017) [114] | 2 (Baseline, 12 months) |  | X |  | X |  |  | X |  |
| Scogin (2007) [110] | 4 (Baseline, mid treatment, end of treatment, 6 months post treatment) | X^short, long^ | X^short, long^ |  | X^short, long^ |  |  |  | X |
| Wang (2022) [111] | 2 (Baseline, 6 months) |  | X^short^ |  |  |  |  | X^short^ |  |
| Wu (2023) [115] | 2 (Baseline, 13 weeks) |  | X^short^ |  |  | X^short^ |  |  |  |
| Xie (2019) [112] | 3 (Baseline, 8 weeks (end of intervention), 3 months post intervention) | X^short^ | X^short^ |  |  |  |  |  |  |

X indicates at least one measure of that outcome is significant

^short^Significant short-term outcome/s

^long^Significant long-term outcome/s

(S) Self report measures were used

(O) Objective measure were used

^*^Anxiety (S) includes worry severity, anxiety symptoms, psychological flexibility and inflexibility, psychological symptomatology

^†^Depression (S) includes depressive symptoms, psychological flexibility and inflexibility, depressive severity, hopelessness, loneliness, major depressive disorder, suicidality, psycholgoical symptomatology, happiness

^‡^Hypertension includes blood pressure

^§^Wellbeing and Quality of life (S) includes health-related quality of life, self-esteem

^||^Self-efficacy (S) includes self-efficacy, resilience, self-perception of aging

^{^Social network (S) includes social network, and social participation attitude

^#^Cognition and function includes functional status, global cognitive function

^**^Other includes level of disability, unmet needs, changes in behavioural activation, functional status
